# Management of ulcerative colitis by dichloroacetate: Impact on NFATC1/NLRP3/IL1B signaling based on bioinformatics analysis combined with in vivo experimental verification

**DOI:** 10.1007/s10787-023-01362-2

**Published:** 2023-10-30

**Authors:** Esraa Abdel-Nassir Abdel-Razek, Heba M. Mahmoud, Amany A. Azouz

**Affiliations:** Pharmacology and Toxicology Department, Faculty of Pharmacy, Beni-Suef University, Beni-Suef, 62514 Egypt

**Keywords:** Dichloroacetate, Ulcerative colitis, Bioinformatics, NFATC1/NLRP3/IL1B signaling, Oxazolone in vivo model

## Abstract

The inflammatory response in ulcerative colitis (UC) could be relieved by the conventional immunomodulatory agents; 5-aminosalicylic acid, corticosteroids, or azathioprine. However, the low remission rates and the intolerance to these agents necessitate investigation of gene expression signature in UC that could influence the therapeutic efficacy of drugs, as well as the interference with persistence genes by novel therapeutic option. Three microarray datasets (GSE66407, GSE38713 and GSE14580) from the NCBI-GEO database were utilized. Differentially expressed genes between samples of patients with UC and healthy ones were analyzed using R software. In addition, in vivo study using oxazolone-induced UC in BALB/c mice was carried out to investigate the proposed therapeutic efficacy of dichloroacetate (DCA). The bioinformatics analysis revealed the persistence of NLRP3, NFATC1, and IL1B in UC despite treatment with common therapeutic agents. DCA administration to oxazolone-treated mice showed remarkable interference with those persistence genes. Western blotting analysis for NLRP3, NFATC1, nuclear/total NF-κB, and cleaved caspase-1 revealed the ability of DCA to reduce the expression levels of these proteins in oxazolone-treated mice. Additionally, the inflammatory cytokines IL-1β and IL-13 were reduced in colonic tissue by DCA treatment. The therapeutic efficacy of DCA was further confirmed by the apparent reduction in histopathological scoring, disease activity index, and the normalization of colon length. Therefore, DCA could be suggested as a novel and promising therapeutic option in UC based on its ability to interfere with the persistence of NFATC1/NLRP3/IL1B signaling. That merits further safety/toxicological pre-clinical assessment and update of bioavailability/metabolism data prior to clinical investigation.

**Supplementary Information:**

The online version contains supplementary material available at 10.1007/s10787-023-01362-2.

## Introduction

Ulcerative colitis (UC) is an idiopathic colonic inflammation that causes widespread friability of the colonic mucosa, superficial erosions, and bleeding. It has been considered as the most prevalent inflammatory bowel disease worldwide. Unfortunately, UC is a lifelong incurable illness with major physical and mental health consequences (Lamb et al. 2019). The pathophysiology of UC is influenced by leukocyte infiltration to inflamed tissues. Further, up-regulation of tumor necrosis factor-alpha (TNF-α), interleukin-13 (IL13), and natural killer T cells occurs in UC. Also, the levels of IgM, IgA, and IgG increase in UC as an inflammatory bowel disease. However, UC patients have a disproportionate rise in IgG1 antibodies (He et al. 2022).

Typically, the emergence of the disorder is subtle, and patients likely endure spontaneous remissions followed by relapses. Treatment protocols include administration of 5-aminosalicylic acid (5-ASA) or corticosteroids that relieve about 50% of the flare symptoms. Despite this progress of treatment, corticosteroids could not be administered for maintenance therapy and 5-ASA has some drawbacks, such as acute intolerance syndrome (diarrhea, fever, abdominal pain) that could result in hospitalization, and colectomy. Further, recent genome-wide association studies and meta-analyses have connected certain single nucleotide polymorphisms (SNPs) to 5-ASA allergy (Kucharzik et al. 2020; Cottone et al. 2011; Chen et al. 2022; Mikami et al. 2023).

The analysis of microarray data reveals the physiological and pathological states of many genes, which aids in the diagnosis and treatment of diseases. Recently, with the improvement of gene sequencing platforms, bioinformatics analyses have been used to identify differentially expressed genes. It has been published a number of reports on the use of arrays and chips for UC bioinformatics analysis (Shi et al. 2020a; Zhang et al. 2020; Huang et al. 2023), but the actual analysis for UC is still relatively little. As a result, bioinformatics techniques could improve our ability to investigate and comprehend UC’s underlying mechanisms.

Dichloroacetate (DCA) is a non-selective inhibitor of mitochondrial pyruvate dehydrogenase kinase. Its clinical utilisation spans over three decades for human hereditary mitochondrial metabolic diseases and congenital lactic acidosis (do Nascimento et al. 2021; Stacpoole et al. 1992, 1997). In recent times, there has been a growing fascination with its anticancer attributes (Tataranni and Piccoli 2019). Besides, DCA exhibits several attributes that render it a promising candidate for several inflammatory diseases, including safety towards healthy cells, remarkable bioavailability, and cost-effectiveness (Mercken et al. 2009; Saghir and Schultz 2002).

The anti-inflammatory effect of DCA has been demonstrated experimentally by Bian et al. (2009) against collagen II-induced arthritis in in female mice. As well, DCA has been reported to significantly inhibit T cell activation and development of airway inflammation in asthma (Ostroukhova et al. 2012). Interestingly, DCA has recently been demonstrated to suppress vascular inflammation and promote plaque stability in atherosclerosis through inhibiting NLRP3 inflammasome activation and IL-1β secretion by macrophages in the plaque (Forteza et al. 2023). The aforementioned anti-inflammatory features of DCA encouraged us to investigate its efficacy as NLRP3 inhibitor and in turn as a possible candidate for UC treatment.

Accordingly, we aimed to investigate the potential pathways and targets for UC treatment using network pharmacology and bioinformatics in our study. It was designed to perform high throughput transcriptome analysis extracted from various datasets of human colon biopsies in healthy, UC, UC-received 5-ASA in both active and remission phases, and also for those patients who are resistant to corticosteroids. Besides, we aimed to verify results of bioinformatics through investigation of the impact of DCA on NFATC1/NLRP3/IL1B signaling using in vivo oxazolone-induced UC model in BALB/*c* mice, providing a beneficial reference to find clinical grade therapeutic option to be added to main therapeutic course of UC.

## Materials and methods

### Acquisition of data of gene expression profiles and transcriptome analyses

Three UC-related data sets. The 1st selected data set (GSE66407) analysis was performed on human samples to compare between UC and normal biopsies. The 2nd selected data set (GSE38713) analysis was performed comparing the transcriptome of active or remission phases of patients receiving (5-ASA), including normal healthy colon transcriptome signature to these comparisons. The 3^rd^ selected data set (GSE14580) analysis was performed to compare between samples of corticosteroids resistant UC patients and those of normal healthy colon. The three data sets were selected from publicly available Gene Expression Omnibus NCBI-GEO (GEO, https://www.ncbi.nlm.nih.gov/geo/) online database of microarray/gene profile. For data visualization, further analyses were performed using R software, online available bioinformatics tools and Graph Pad Prism V. 9. Negative logarithmic 10 of adjusted *P*. values cut-off was set at 1.15 (Adj. *P* value = 0.05). False discovery rate (FDR) was used for statistical and multiple comparisons analysis.

### Mice strains, housing, and induction of oxazolone colitis

Male BALB/c mice at 6–10 weeks of age purchased from Theodor Bilharz Research Institute (Giza, Egypt) have been utilised in the experimental model of oxazolone-induced colitis. Animals were housed 10 days for adaptation under Murine Pathogen Free conditions including room temperature 22 ± 2 °C, while relative humidity was about 55% ± 10%, and lighting interval 7.00 A.M.–7.00 P.M. Food as Chow pellet and untreated water were available at libitum.

Acute ulcerative colitis was induced in BALB/c mouse strain that are susceptible to Th2 immune activation as previously described (Siregar and Widyawati 2021; Wirtz et al. 2007). That was initiated by pre-sensitization of mice through percutaneous application of 150 μL 3% oxazolone (4-ethoxymethylene-2-phenyl-2-oxazolin-5-one, CAT# 862207, Sigma-Aldrich, St Louis, MO, USA) solution at a dilution (4:1 in acetone and organic extra virgin olive oil) on the animal’s 2 × 2 cm field shaved abdomen. 150 μL oxazolone solution (1%) dissolved in 50% ethanol was used for challenge on day 5 by intra-rectal administration using 18-gauge stainless steel gavage needle, 4 cm depth into the colon, followed by holding the mice vertically for 30 s. DCA (CAT# 347795, Sigma-Aldrich, St. Louis, MO, USA) was administered orally at a dose of 100 mg/kg 1 h before instillation of oxazolone enema and for 3 subsequent days.

### Disease activity assay

Throughout the course of treatment, body weight change, the presence and viscosity of blood in stool were observed and recorded on a daily basis. The severity index which also known as the disease activity index (DAI) was calculated as the sum of the scores for body weight loss (0: < 1%, 1: 1–5%, 2: 6–10%, 3: 10–15% and 4: > 15), stool consistency (0: normal pellets, 1: slightly loose feces, 2: loose feces, 3: watery diarrhea) and rectal bleeding (0: no rectal bleeding, 1: hemoccult-positive, 2: bloody, 3: heavy bleeding) (Zhou et al. 2022; Shi et al. 2020b). Execution was done for mice on the third day post-challenge. Colon length was carefully photographed and estimated through measuring the distance between the colorectal junction and the end of the distal rectum. The distal segments of the colonic tissues were gathered for diverse assessments, such as microscopic examination and histopathological analysis with hematoxylin and eosin (H&E) staining, ELISA, and Western blot analysis.

### Colon histopathological investigation

For hematoxylin and eosin (H&E) staining, briefly, the isolated distal colonic tissues of mice were fixed for 24 h in 4% paraformaldehyde prior to paraffin embedding. After dehydration of the samples, the colon tissue was sliced (4 μm), placed on glass slides, and stained with H&E or Alcian blue at room temperature in a blinded manner to eliminate the possibility of observer bias, in accordance with the criteria that were defined and explained before (Bancroft and Gamble 2008; Erben et al. 2014). Lamina propria structure and epithelial damage were examined at 200 × magnification using the light microscope (Leica Microsystems, Germany). Scoring for colonic epithelium injury and inflammatory cell infiltration was carried out in line with what is provided in Table 1 according to a microscopic scoring system (Wirtz et al. 2007).

**Table 1:** Histopathological scoring

Score	Histologic changes
0	No evidence of inflammation
1	Low level of inflammation with scattered infiltrating mononuclear cells (1–2 foci)
2	Moderate inflammation with multiple foci
3	High level of inflammation with increased vascular density and marked wall thickening
4	Maximal severity of inflammation with transmural leukocyte infiltration and loss of goblet cells

### Myeloperoxidase (MPO) and nitric oxide (NO) activity assay

Myeloperoxidase (MPO) enzymatic activity in colon tissue was assessed bio-chemically as stated previously (Manktelow and Meyer 1986). This was accomplished by spectrophotometrically detecting the color change at 460 nm that occurred in a reaction cocktail because of dimethoxybenzene being oxidized via the in situ produced H_2_O_2_. Determination of NO content in colon tissue was done as previously described (Giustarini et al. 2008).

### Enzyme-linked immunosorbent assays for IL-1β and IL-13 in colon tissue

Colonic inflammatory cytokine concentrations of IL-13 (CAT# BEK1121, Chongqing Biospes Co., Ltd, China), and IL-1β (CAT# E-EL-R0019, Elabscience Biotechnology Inc., USA) were assessed using ELISA kits following the instructions provided by the manufacturer. Shortly, the procedure involved combining the colon tissue homogenates with purified mice IL-13 and IL-1β antibodies, which were then coated onto microliter plates. Following several washes, (TMB) substrate solution was added to produce a blue color following reaction with horseradish peroxidase. After 15 min, the stop solution was added and the produced yellow color was determined by utilizing a Robonik Readwell ELISA reader (India) at 450 nm.

### Western blotting analysis

Total protein of the distal colonic tissue samples was extracted by homogenization in RIPA lysis buffer, with added protease inhibitor and phosphatase inhibitor (Biospes, China) at a temperature of 4 °C for 30 min. The upper clarified lysate was collected subsequent to centrifugation at 15,000 rpm for 15 min at 4 °C. The total protein concentration of each sample had been assessed using a BCA kit (Beyotime Biotechnology) as explained by the Biuret method (Towbin et al. 1979). For Western blotting, 20 μg sample of the extracted protein of each group was isolated by 10%–12% SDS-PAGE and transferred to a polyvinylidene fluoride membrane (Millipore, Merk, USA) via semidry methods (Wang et al. 1996). The membrane was blocked with 5% BSA solution in Tris-buffered saline and 0.1% Tween-20. The membrane was then incubated with specific primary antibodies including anti-NLRP3 (CAT# YPA1480, Chongqing Biospes Co., Ltd, 1:1000), anti-NF-κB (CAT# abx012874, Abbexa LTD, Cambridge, UK, 1:500), anti-cleaved caspase-1(CAT# YPA2348, Chongqing Biospes Co., Ltd, 1:1000), anti-NFATC1 (CAT# YPA2464, Chongqing Biospes Co., Ltd, 1:1000), and anti-β-actin (CAT# sc-47778, Santa Cruz, USA; 1:1000) overnight at 4 °C (12 h). That was followed by washing the membrane with 1 × PBST and incubation with secondary antibodies (Beyotime Biotechnology) at room temperature for 1 h. Next, it was incubated with an enhanced chemiluminescent substrate (Beyotime Biotechnology). Relevant bands were semi-quantified using image J^®^ software (National Institutes of Health, Bethesda, USA).

### Ethical considerations

Experimental protocols were conducted in accordance with guidelines of the National Institutes of Health Guide for the Care and Use of Laboratory Animals. Permission for the project was granted by the Institutional Animal Care and Use Committee (IACUC), Beni-Suef University (IACUC-BSU: 022-339).

### Statistical analysis

The data are expressed as mean ± standard error of the mean (SEM). Significant difference between groups was investigated using one-way analysis of variance (ANOVA) followed by Tukey’s. All statistical analysis and subsequent graphics generation were performed using GraphPad Prism version 9.0 (GraphPad Software, USA). A *P* value < 0.05 was statistically significant.

## Results

### NLRP3, NFATC1, and IL1B showed higher expression levels in human UC biopsies

**Fig. 1:**
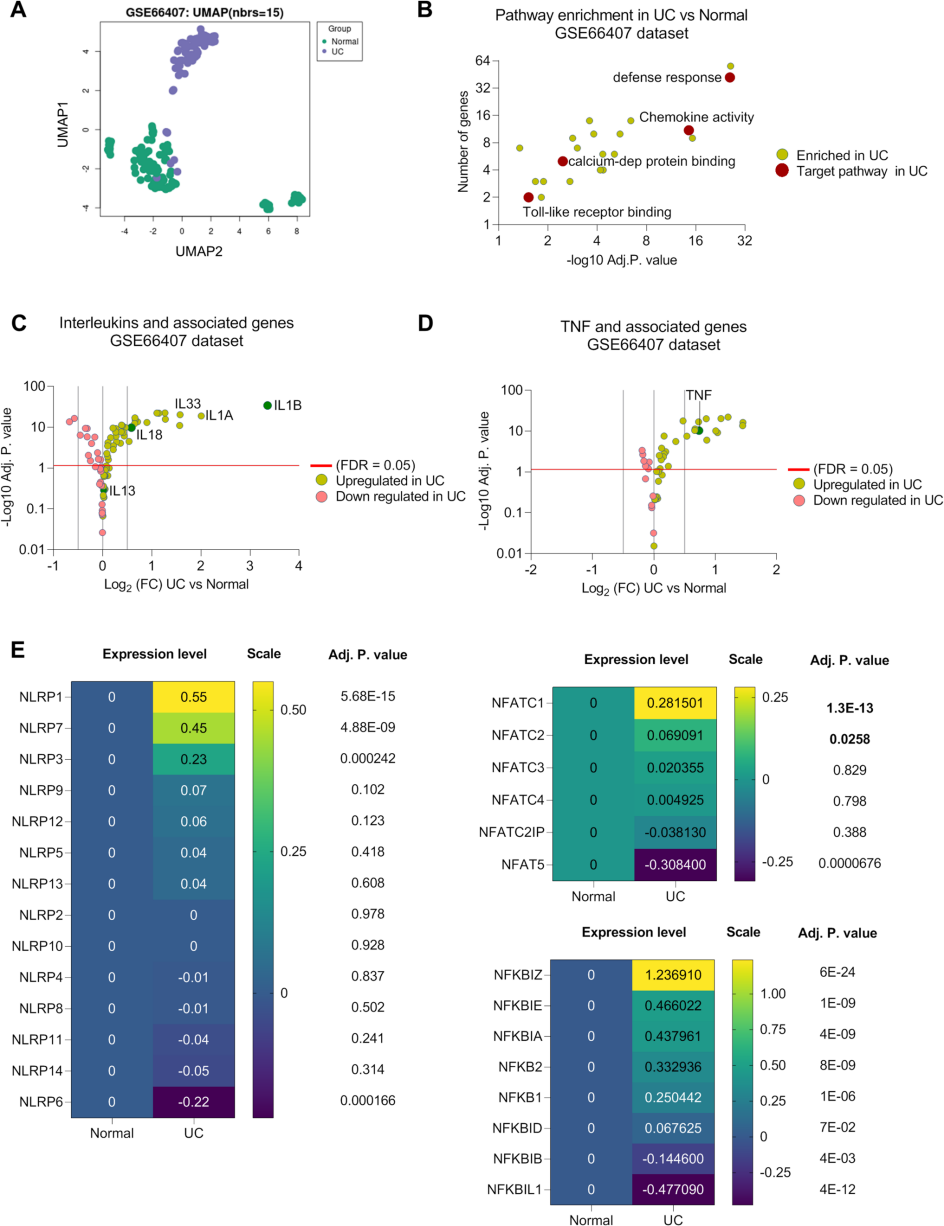
Enhanced expression of specific immunophenotypic response genes in human UC.** A** UMAP scatter plot comparing between UC and normal biopsies as extracted from GSE66407 transcriptome data set. **B** Functional enrichment analysis in UC samples extracted from GSE66407 dataset. Top enriched pathways were selected based on genes with fold change ≥ 3 in UC compared to normal biopsies. The analysis was performed using: Profiler and top 21 enriched pathways were selected for visualisation. Fold changes in expression levels of **C** interleukins and associated genes and **D** TNF and associated genes, in UC versus normal samples extracted from GSE66407 data set. Negative logarithmic 10 of adjusted *P* values cut-off was set at 1.15 (Adj. *P* value = 0.05). False discovery rate was used for statistical and multiple comparisons analysis. **E** Absolute expression levels of NLRP, NFATC1 and NF-κB gene isoforms comparing between Log_2_ fold changes differences between “normal versus normal” and “UC versus normal”

To study the molecular signature changes in UC in human patients, we tended to analyse publicly available GSE66407 transcriptome data set containing biopsies collected from gut tissues, including ascendens, bulbus duodenum, coecum, descendens, ilium, rectum, sigmoideum, and traversum. The transcriptome was profiled for 99 biopsies collected from 28 healthy or normal human donors (group 1) and 60 gut biopsies from 40 inflamed or being active ulcerative colitis patients (group 2). Results showed distinct expression pattern of whole transcriptome of UC and normal biopsies (Fig. 1A). Further, we ran pathway enrichment analysis on significantly top expressed genes showing two-fold higher expression in UC compared to normal counterparts. We further selected top 21 significant enriched pathways. The analysis resulted in significant enrichment of defense response, chemokine activity, and Ca^2+^ dependent protein binding in UC compared to normal colon tissues (Fig. 1B). We further analyzed single transcripts of interleukins (ILs) and TNF-α isoforms and their related genes. Results showed that IL1B, IL1A, IL33, and IL18 expressions were higher in UC samples compared to normal counterparts (Fig. 1C). IL13, however, did not show a significant difference in UC as compared to normal colon tissues (Fig. 1C). TNF-α expression level was higher in UC as expected (Fig. 1D). We further analyzed expression profile of various NLRPs inflammasome isoforms. The analysis highlighted significant increase in NLRP1, 7, and 3 isoforms expression compared to normal colon tissues (Fig. 1E). Next, we proposed possible connection between Ca^2+^-binding proteins and formation of inflammasome in UC. We particularly investigated the expression levels of NFAT isoforms, because NFAT has been reported to enhance NLRP3 activity in human tissues (Shih et al. 2008; Perera et al. 2018). The results showed higher expression of NFATC1 and NFATC2 in UC compared to normal tissue indicating possible role of these proteins in NLRP3 activity in UC (Fig. 1E). Overall, transcriptome analysis of biopsies collected from UC patients proposed that inflammation-driven symptoms could potentially rely, among others, on NLRP3, NFATC1, and IL1B as revealed through their higher expression levels in UC compared to their corresponding expressions in normal individual colonic biopsies.

### Persistence of NLRP3, NFATC1, and IL1B expression in active phase of UC with history of 5-ASA or corticosteroids treatment

**Fig. 2:**
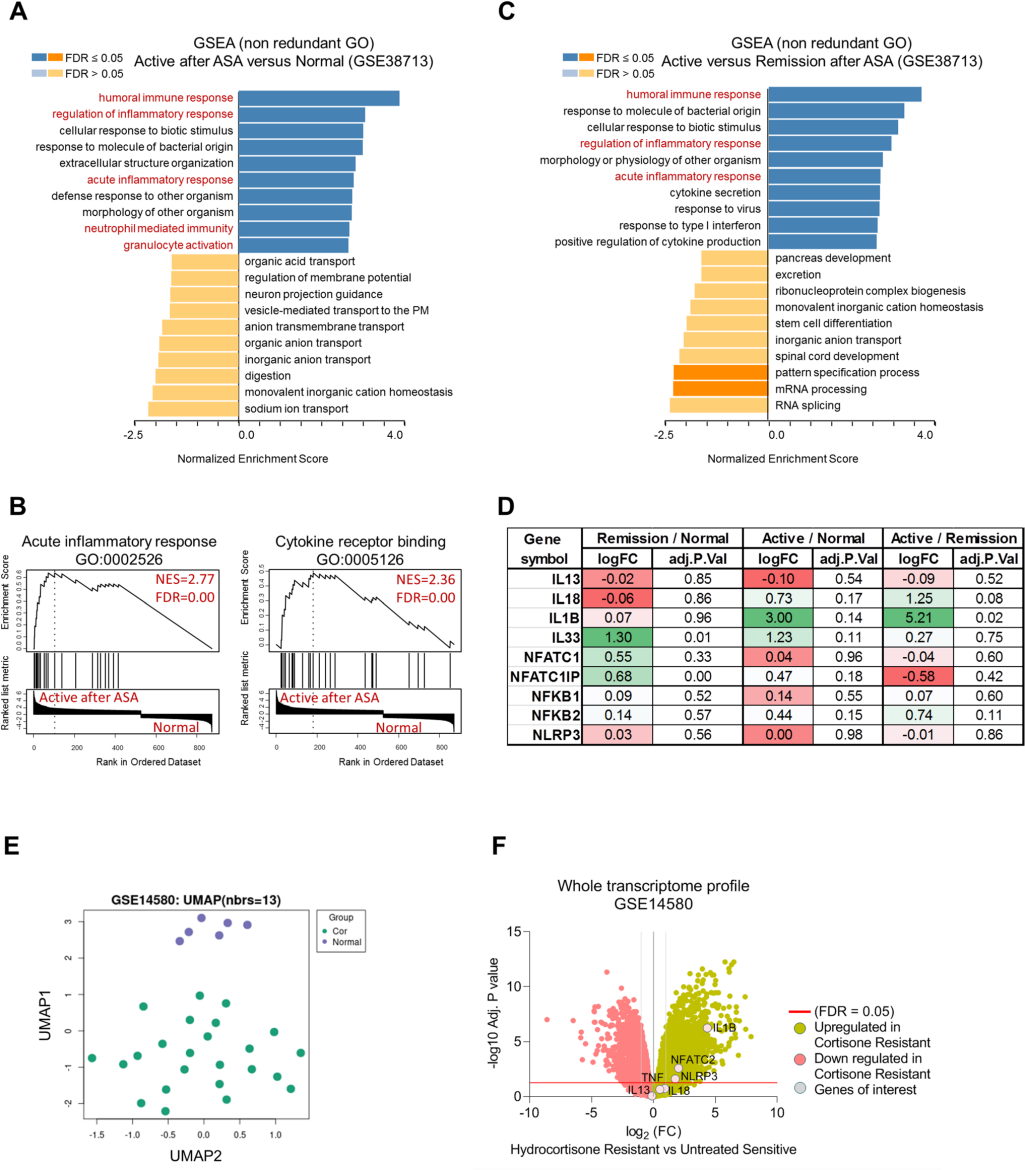
Corticosteroids and immunosuppression resistant UC showed persistence of NLRP3, IL1B, and NFATC1&2 gene expression. **A** Functional analysis of top and down enriched gene lists in active UC after receiving 5-ASA versus normal biopsies as extracted from GSE38713 dataset. The analysis was performed using gprofiler2 R package. **B** Enrichment score of acute inflammatory response and cytokine binding in active UC after 5-ASA versus normal biopsies. Ranking was performed using GSEABase bioconductor R package. **C** Functional analysis of top and down enriched gene lists in active UC after receiving 5-ASA versus normal biopsies as extracted from GSE38713 dataset. The analysis was performed using gprofiler2 R package. **D** Fold expression changes of selected genes in various UC phases of the disease as extracted from GSE38713 using DESeq2 package. **E** UMAP scatter plot comparing between corticosteroids resistant UC patients and those of normal healthy colon samples as extracted from GSE14580 transcriptome data set. **F** Fold changes in whole transcriptome comparing between corticosteroids resistant UC patients and those of normal healthy colon samples as extracted from GSE14580 transcriptome data set. Negative logarithmic 10 of adjusted *P* values cut-off was set at 1.15 (Adj. *P* value = 0.05). False discovery rate was used for statistical and multiple comparisons analysis

Given that UC disease symptoms are recurrent episodes of active followed by remission phases, we compared the transcriptome of active or remission phases of patients receiving 5-ASA, included normal healthy colon transcriptome signature to these comparisons. We selected GSE38713 dataset that matches these criteria. We first analyzed gene ontology including significant top and down-regulated genes at least one-fold expression change in active state of the disease compared to normal biopsies. Results showed that active phase of the disease still has enriched humoral immune response, and acute inflammatory response compared to normal tissues (Fig. 2A, B). Further, analysis showed that active state of the disease has enriched cytokine receptor binding as compared to their corresponding normal tissues (Fig. 2B). We applied similar approach on same dataset to compare between pathway enrichment in active and remission phases of the disease (Fig. 2C). Analysis revealed consistent enrichment of inflammatory and immune response in active status as compared to enrichment levels in remission phase. We further selected those genes that we proposed to participate in the inflammation process and cytokine-mediated signaling in either active, remission phase of the disease and those of normal tissues (Fig. 2D). Results showed that the NLRP3 expression level does not change despite 5-ASA treatment in active, remission, and normal tissues, proposing persistence expression of inflammasome components despite 5-ASA treatment. Further, the analysis showed that, among all relevant cytokines, only IL1B showed significant expression in active phase of the disease, proposing possible role of higher expression of IL1B in driving cytokine-mediated signaling in active phase of the disease despite 5-ASA treatment. Further, NFATC1 showed non-significant changes in different disease phases as compared to normal tissues, indicating lack of 5-ASA effect on altering the expression level of NFATC1 in UC. In addition, NFATC1 interacting protein (NFATC1IP), which is known to enhance nuclear transcription activity of NFATC1, showed higher expression in remission phase as compared to normal levels, indicating possible role in enhancing NFATC1 activity in remission phase which may participate in late disease relapse. However, this requires further and throughput investigation.

We next aimed to compare NLRP3, IL1B, and NFATC1 expression levels in GSE14580 transcriptome data set comparing between colon biopsies collected from 24 patients with active UC diagnosed as refractory to corticosteroids and/or immunosuppression, and biopsies collected from another 6 normal individuals. Results showed that corticosteroids resistant UC samples have higher expressions of NLRP3, IL1B, and NFATC1 as compared to normal tissues (Fig. 2E and F).

Overall, both transcriptome analyses of samples collected in active phase of UC despite 5-ASA treatment, and those who are resistant to corticosteroids proposed that NLRP3, IL1B, and Ca^2+^-binding protein NFATC1 could participate in the transcriptional activity, cytokine signaling, inflammasome formation in 5-ASA or corticosteroids refractory UC patients.

### DCA significantly reduced expression levels of NLRP3, NFATC1, nuclear NF-κB, and cleaved caspase-1 in oxazolone-induced colitis mouse model

**Fig. 3:**
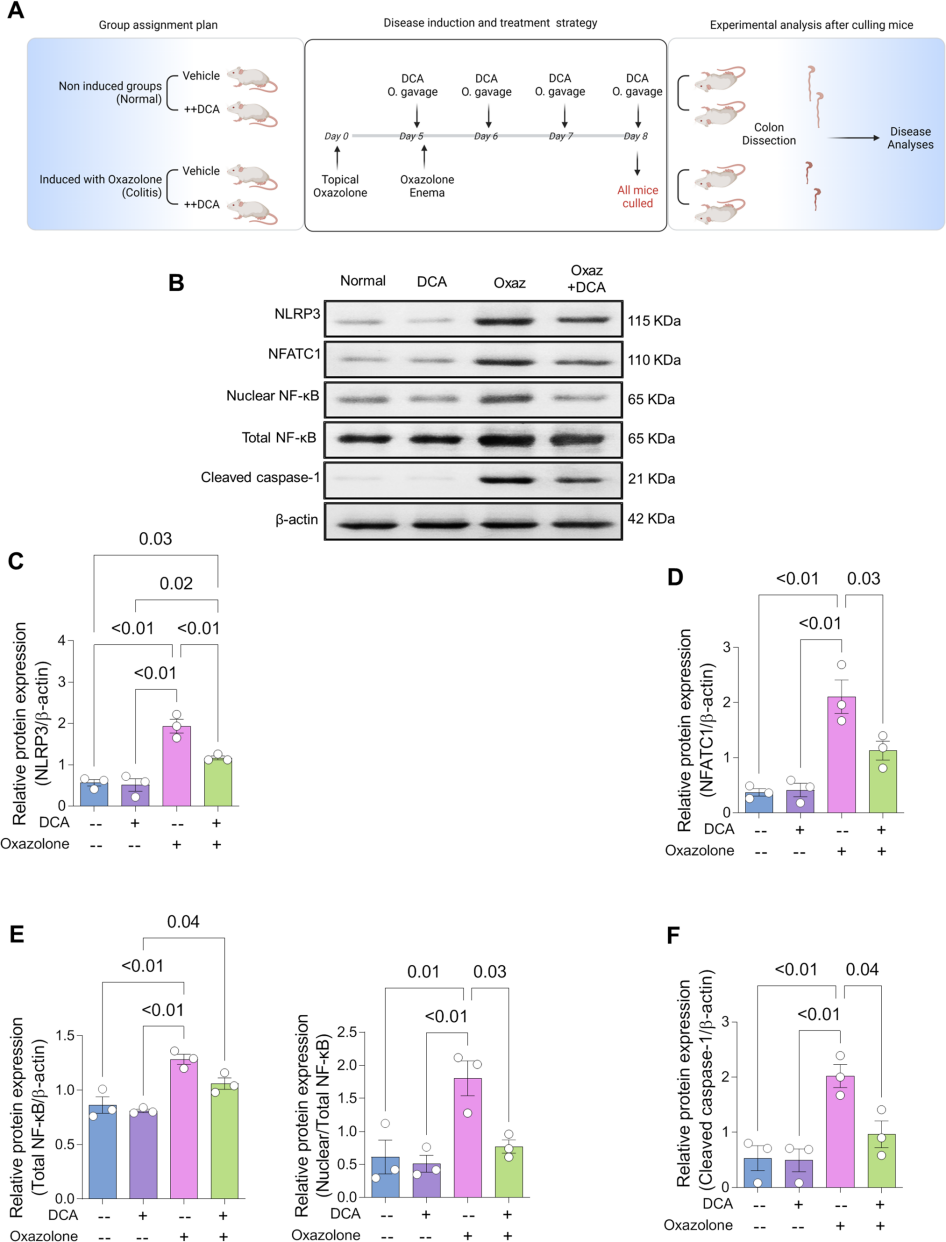
DCA treatment attenuated NLRP3, NFATC1, nuclear NF-κB, and cleaved caspase-3 expression levels in colitis-induced mouse model.** A** Schematic diagram illustrating colitis disease induction using oxazolone and treatment strategy using DCA in BALB/c mouse model (UC mouse model). **B** Representative Western Blot indicating protein levels in colons dissected from normal and UC mice received vehicle or DCA treatment. The relative protein expressions of **C** NLRP-3, **D** NFATC1, **E** Total and nuclear NF-κB, and **F** cleaved caspase-1 in colons dissected from normal and UC mice received vehicle or DCA treatment (*n* = 3 mice for each group). For (**C**–**F**), means ± S.E.M. *P* values were calculated using one-way ANOVA and Tukey’s test for multiple comparisons correction

Next, we aimed to investigate if targeting the expression level of NLRP3, IL1B, and NFATC1 could alleviate symptoms of UC. To deliver this approach, we selected the chemical compound DCA based on literature mining. We selected DCA, because it has been reported to inhibit NLRP3 activation (Berg 2020; Forteza et al. 2023). Next, we established chemically induced colitis model using oxazolone as previously reported in BALB/c mice (Siregar and Widyawati 2021) followed by testing the effect of DCA on alleviating oxazolone-induced colitis. Colitis was induced in BALB/c mice using topical 3% oxazolone solution (day 0) followed by enema of 1% oxazolone solution dissolved in ethanol (day 5). Mice received daily dose of 100 mg/kg DCA by oral gavage for 4 consecutive days starting on the same day of enema oxazolone administration. To investigate the effect of DCA, mice were culled on the last day of DCA administration (day 8) followed by dissecting colons from normal group that did not get induced for colitis or received DCA, normal group received DCA, colitis group that did not get treated with DCA, and lastly colitis group treated with DCA (Fig. 3A). Tissues were homogenised and proteins were extracted for analysis using Western Blot technique. Results showed that DCA treatment in normal mice did not alter the expression level of NFATC1, NLRP3, nuclear fraction of NF-κB, or cleaved caspase-1 (Fig. 3B–F). Further, it is known that NLRP3 inflammasome participates in the activation of pro-caspase-1 cleavage into the active form of caspase-1 (Itani et al. 2016; Shih et al. 2008). Based on that, we measured if DCA-induced reduction in the expression level of NLRP3 would reduce the level of cleaved caspase-1 and hence IL-1β level in oxazolone-induced colitis as measured by Western Blot. DCA significantly reduced the level of cleaved caspase-1 by 50% in oxazolone-induced colitis, indicating effective reversal of caspase-1 activation in colitis through reducing NLRP3 protein level (Fig. 3B, C, F). The activity of NLRP3 could be lowered in response to reduction in Ca^2+^-binding protein level of NFATC1 (Itani et al. 2016; Martínez-Martínez et al. 2006; Pan et al. 2013; Shih et al. 2008) which is required for several gene transcriptions that could participate in NLRP3 activation. Our results revealed that DCA significantly reduced the level of NFATC1 by 50% and NLRP3 by 40% in oxazolone-induced colitis (Fig. 3B, C, D). Another dimension that could reduce NLRP3 inflammasome activation was through DCA-induced reduction in nuclear translocation of NF-κB which is known to participate in NLRP3 activation. Western blot analysis in our study showed markedly reduced expression level of nuclear NF-κB by 50% (Fig. 3B, E). Overall, DCA was able to reduce NLRP3 protein level indicating either possible enhanced degradation or interference with its gene expression. Beside the ability of DCA to reduce NLRP3 level (Berg 2020; Forteza et al. 2023), it also reduces protein levels that could participate in NLRP3 activation, such as nuclear fraction of NF-κB (Kan et al. 2018) and NFATC1 (Guignabert et al. 2009). Further, the NLRP3-mediated inflammasome activity was inhibited based on results showed that DCA was able to reduce expression level of cleaved caspase-1 and inhibit the activation of IL-1β in colitis-induced mouse model (Forteza et al. 2023).

### DCA significantly reduced inflammatory and oxidative stress biomarkers in oxazolone-induced colitis model

**Fig. 4:**
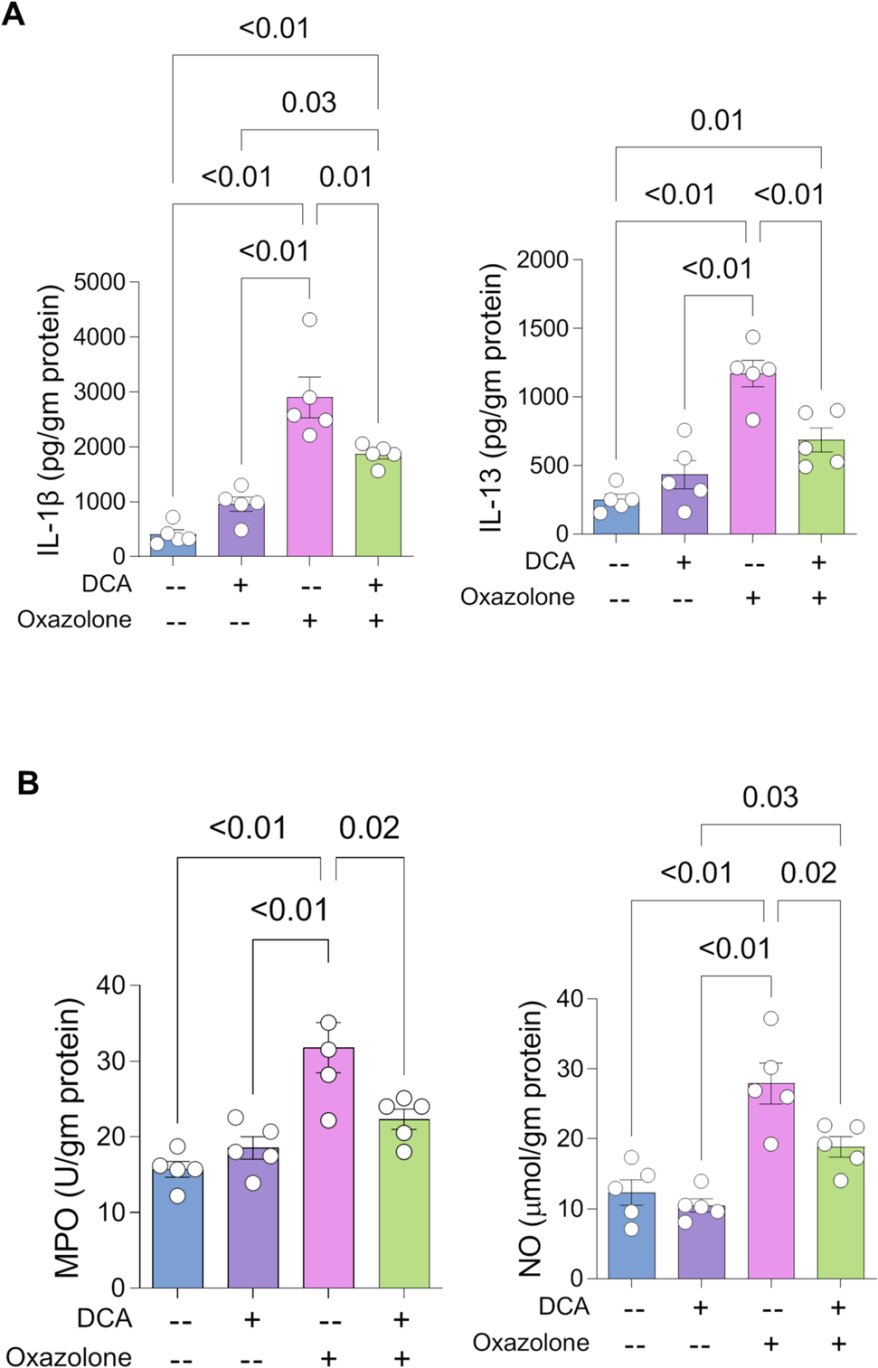
DCA treatment attenuated interleukins levels and oxidative stress of colitis-induced mouse model. **A** Levels of IL-1β and IL-13 in colons dissected from normal and UC mice received vehicle or DCA treatment (*n* = 5 mice per assigned group). **B** Spectrophotometric analysis of MPO activity (units/gm protein) in colons dissected from normal and UC mice received vehicle or DCA treatment (*n* = 5 mice for each group). For (**A** and **B**), means ± S.E.M. *P* values were calculated using one-way ANOVA and Tukey’s test for multiple comparisons correction

The reduction in expression level of NLRP3 and its activity was further validated by measuring IL-1β, IL-13, MPO, and NO (Itani et al. 2016; Bauer et al. 2012) in colitis-induced mouse model. ELISA and biochemical analysis were performed as previously reported and results showed that DCA significantly reduced IL-1β by 36% (Forteza et al. 2023), IL-13 by 40% (Ostroukhova et al. 2012) (Fig. 4A), and NO levels by 30%, as well as MPO activity by 30% (Saed et al. 2011; Torres-Cavazos et al. 2020) (Fig. 4B). Another dimension of DCA action on inflamed colon where DCA significantly reduced the level of oxidative stress, indicating potential ability of DCA to reduce genetic aberration and tissue damage resulting from enhanced oxidative stress. Further, oxidative stress reduction could result from reduced cytokine induced inflammation.

### DCA treatment improved colonic morphology and retarded weight loss and disease activity of colitis-induced mouse model

**Fig. 5:**
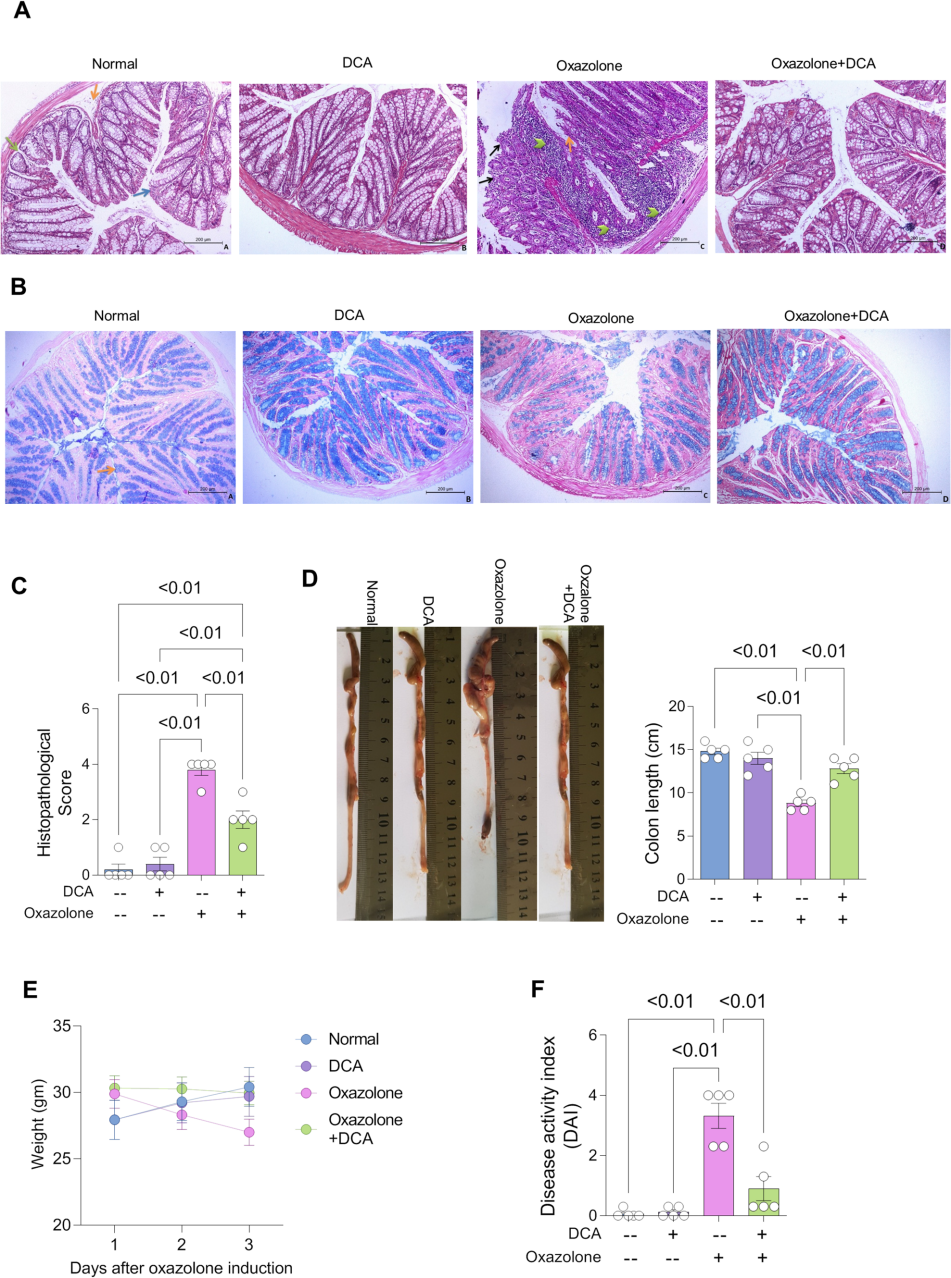
DCA treatment improved colonic morphology and retarded weight loss and disease activity of colitis-induced mouse model.** A** Representative photomicrographs demonstrating colonic sections (× 200) following H&E-staining of colons dissected from normal and UC mice received vehicle or DCA treatment. As normal group showed normal histological structure of the colon and regular arrangement of the lining layers; mucosa (blue arrow), submucosa (orange arrow) and musculosa (green arrow). As well, the normal group received DCA showed normal histopathological features. While colitis group revealed the histopathological features of colitis represented by mucosal hyperplasia (orange arrow), massive focal to diffuse infiltration of mononuclear cells and neutrophils in the lamina propria and submucosa (arrow head), and mucosal ulcerations (black arrow) in some areas were detected in the colon. Colitis group treated with DCA showed marked improvement of colitis pathology in the form of normal preserved histological colon wall structure, but mild inflammatory cell infiltrations in the lamina propria were still observed. **B** Representative photomicrographs demonstrating colonic sections (× 200) following Alcian blue staining to determine goblet cells of colons dissected from normal and UC mice received vehicle or DCA treatment. Normal group showed intact Alcian blue positive goblet cells in the epithelium and crypts lining (arrow), normal group received DCA revealed abundant goblet cells, while colitis group showed marked reduction in goblet cells, but colitis group treated with DCA showed an almost normal content of goblet cells in colonic section. **C** Histopathological score following H&E staining (*n* = 5 mice for each group). **D**–**F** Images showing dissected colons from mice receiving vehicle or DCA, mice weights after oxazolone administration and disease activity index in those mice

Based on results that DCA reduced the inflammatory response of NLRP3 inflammasome and oxidative stress in oxazolone colitis model, we tended to score DCA impact on DAI and the integrity of colon tissue using histopathological colon sectioning and staining, followed by colon ulceration scoring. Results showed that DCA did not neither alter DAI and colon length nor increase histopathological score when compared to normal healthy colon, proposing to consider DCA as a safe drug choice that did not show any deterioration of normal colon tissues (Fig. 5A–C). Further, DCA significantly retarded oxazolone enhanced DAI by 60%, ulceration scoring, and preserved colon length by 40%, indicating the effectiveness of DCA for treating colitis in vivo (Fig. 5C, D, F). Moreover, DCA significantly ameliorated colitis-induced body weight loss, probably by ameliorating oxazolone-induced anorexia through treatment period (Fig. 5E).

## Discussion

Millions of individuals around the world suffer from ulcerative colitis, a form of inflammatory bowel disease marked by weight loss, diarrhea, rectal bleeding, and abdominal discomfort that is notoriously difficult to be treated, easy to return, and predisposed to cancerization. The therapeutic objectives for ulcerative colitis have been shifted from symptom management to mucosal healing in recent years. This is due to the disease’s poorly understood pathophysiology as well as its potentially disabling and progressive devastating course. However, traditional therapies including 5-ASA and corticosteroids or other immunosuppressive drugs such as azathioprine are mostly limited to controlling inflammation and clinical symptoms, and it is difficult to achieve the expectations of long-term maintenance (Chen et al. 2022).

Biological technology has revealed various UC biomarkers for early detection and treatment. If we tackle genetics, cytokine abnormalities, or microbiota in the colon, we can improve therapeutic outcomes (Goyette et al. 2007; Axelrad et al. 2016; Taku et al. 2020). We selected the GSE66407, GSE38713 and GSE14580 transcriptome data sets for an in-depth analysis of biological information and to study the molecular signature changes of UC in human patients. Transcriptome analysis of biopsies collected from UC patients with inflammation-driven symptoms could potentially rely, among others, on NLRP3, NFATC1, and IL1B as confirmed by their higher expression levels in UC in active or remission state even after treatment with 5-ASA compared to their corresponding expressions in normal individual colonic biopsies.

Therefore, we established UC model in mice by using oxazolone as an inducing agent in BALB/c mice, which has been approved to be the most model that resembles human UC (Baydi et al. 2021; Siregar and Widyawati 2021). Through oxazolone-induced UC model, we validated the highest transcribed genes level in the first cluster of colonic tissues and their related cytokines using Western blotting analysis and ELISA to verify the main pathophysiological roles of these core genes. The expression of the NLRP3, NFATC1, as well as IL-1β; the prominent key cytokine that provokes the inflammatory response in colitis and the most likely downstream effector molecule of the NLRP3 inflammasome were significantly elevated in the colitis model in harmony with our bioinformatics analysis.

Our in vivo study was established to investigate the potential ameliorative effect of DCA standing against oxazolone colitis and its effect on NLRP3, NFATC1, and IL-1β as the predominant mediators of UC according to results of the bioinformatics analysis.

Consistent with previous studies, oxazolone-induced colitis was evidenced by extreme body weight loss, significant decline of colon length, diarrhea, and rectal bleeding with respect to several histological alterations in mice (Siregar and Widyawati 2021; Boirivant et al. 1998). The present investigation demonstrated that DCA administration markedly reduced leukocyte infiltration into the inflamed colon subsequent to oxazolone instillation as demonstrated by the histopathological evaluation, and the significant restoration of colon length and DAI scores.

According to previous reports examining human tissue and animal models, UC is characterised by significant infiltration of inflammatory cells, particularly activated T lymphocytes, contributing to the disease's pathogenesis. The majority of patients with UC have cytokine profiles characteristic of type 2 helper T (Th2) cells, such as IL-4, IL-5, and IL-13. (Heller et al. 2002; Dohi et al. 1999). Shih et al. (2008) have investigated the colonic tissue sections obtained from patients with UC, Crohn’s disease (CD), cases of non-specific colitis, and cases of normal controls. The findings revealed that all of UC patients exhibited an up-regulation of NFATC1 expression in the colonic mucosa, which was largely attributed to the escalation in the number of mucosa infiltrating lymphocytes and the up-regulation of NFATC1 in each infiltrating lymphocyte.

Multiple lines of evidence (Lin et al. 2022; Shih et al. 2008; Elloumi et al. 2012) have documented that nuclear translocation and activation of NFATC1 in infiltrating lymphocytes in colonic mucosa affected by UC as well as the degree of nuclear translocation/activation of NFATC1 is strongly correlated with disease activity, as indicated by clinical and histological grading. This suggests that NFATC1 plays a direct role in the pathogenesis of UC. It is worth noting that this phenomenon is specific to UC, as it is relatively uncommon in CD and non-specific colitis. Furthermore, the activation of NFATC1 induces the transcription of NLRP3 inflammasome which in turn stimulates the transcription of various pro-inflammatory cytokines, thus aggravating the inflammatory response (Zhu and Cao 2017). In addition, the activation of NFATC1 is primarily associated with the commitment of Th2 differentiation, that promotes secretion of IL-4, which is rapidly superseded by IL-13 production (Wang et al. 2006; Shih et al. 2008). Given their importance, IL-13 is one of the main cytokines responsible for initiating the detrimental inflammatory cascade in UC, influencing epithelial cell function with subsequent disruption of the intestinal epithelial barrier and increased severity of the disease, leading eventually to apoptosis (Heller et al. 2002; Hoving et al. 2017).

In harmony with previous studies, our results showed significant up-regulation of NFATC1 expression with higher histopathological scores in mice with oxazolone-induced colitis (Elloumi et al. 2012; Pan et al. 2013), in addition to the induced extensive inflammatory response evidenced by enhanced IL-13 production (Heller et al. 2002). Meanwhile, DCA treatment exhibited prominent ameliorative effect on oxazolone-induced inflammatory response as evidenced by significantly reduced NFATC1 expression and IL-13 level, which in accordance with other studies (McMurtry et al. 2004; Guignabert et al. 2009).

Nuclear factor-kappa B, a crucial transcription factor that is implicated in UC pathogenesis, has emerged as the main regulator of the intestinal inflammation associated with active UC. As reported by previous researches via using immunofluorescence assays, NF-κB activation occurs primarily in the intestinal macrophages and mucosal epithelial cells of the intestinal lumen (Lu and Zhao 2020; Atreya et al. 2008). Notably, a positive correlation has been identified between the number of cells exhibiting activated NF-κB and the severity of intestinal inflammation. In intestinal macrophages, NF-κB stimulates transcription of the pro-inflammatory cytokines such TNF-α and IL-1β, which are crucial for initiating and sustaining intestinal inflammation in individuals with active UC, moreover, they have a direct role in mediating mucosal tissue injury by, primarily, activating the synthesis of matrix metalloproteinases (Wang et al. 2018).

Apart from that, the activation of NF-κB triggers transcription of the NLRP3 inflammasome, characterised as a substantial protein complex composed of NLRP3, adaptor apoptosis-associated speck-like protein containing a CARD domain (ASC), and pro-caspase-1. The NLRP3 protein is primarily activated within macrophages, which serve as tissue sentinels. The activation of macrophages is crucial in the restoration of innate immune responses, given that M1 macrophages are known to produce inflammatory cytokines and nitric oxide, which are potent promoters of inflammation (Kanneganti and Lamkanfi 2007). The activation of NLRP3 involves two distinct signals. The first signal entails NF-κB activation, driven by the pathogen-associated molecular pattern (PAMP), which triggers the production and storage of precursor proteins, including NLRP3 and pro-IL-1β, while the second one involves the damage-associated molecular pattern (DAMP) triggered NLRP3 activation. The later signal recruits pro-caspase-1 through the linker protein ASC, leading to self-catalyzed processes and formation of activated caspase-1 that cleaves pro-IL-1β and pro-IL-18 into their mature forms, which are released as pro-inflammatory cytokines, starting most inflammasome-derived inflammatory reactions (Itani et al. 2016). Cleaved caspase-1 promotes programmed cell death which negatively impacts the mucosal barrier integrity by translocating and rupturing mucosal cells, releasing massive amounts of pro-inflammatory cytokines to activate immunological mediators like IL-1β and IL-18. That in turn also plays a crucial role in stimulating the phosphorylation of NF-κB and the activation of NLRP3, thus aggravating the inflammatory response (Tourkochristou et al. 2019; Guo et al. 2015; Petrilli et al. 2005). Moreover, Bauer et al. (2012) have verified that NLRP3-deficient mice exhibited substantial protection against the development of colitis. These reports are consistent with our findings from bioinformatics analysis that NF-κB/NLRP3/IL-1B axis may represent possible therapeutic target for patients who suffer from inflammatory bowel disease in the future.

Results of our in vivo study consistent with previous reports (Ullah et al. 2022; Feng et al. 2012; Itani et al. 2016; Zherebiatiev and Kamyshnyi 2016) and our bioinformatics analysis demonstrated a marked up-regulation of the inflammatory status with increased expression of NF-κB, NLRP3, cleaved caspase-1 and significant elevation of colonic IL-1β in mice subjected to oxazolone-induced colitis. On the other hand, our findings showed that treatment with DCA successfully revoked the deleterious inflammatory response induced by oxazolone through significantly down-regulated colonic expression of NF-κB, NLRP3, cleaved caspase-1, and reduced IL-1β level. That could certainly attenuate the immunological cascade seen in UC and afford a tentative guide for the anti-inflammatory defensive effect of DCA in UC, which is parallel to other studies (Forteza et al. 2023; Ostroukhova et al. 2012; Bian et al. 2009).

When the intestinal epithelial barrier is disrupted, immune cells become exposed to bacterial products which set off a series of events that activate the mucosal immune system and cause significant inflammation. That can be emphasized by the significant elevation in MPO enzymatic activity; an indicator of neutrophil infiltration; in colonic tissues of patients with active UC (Garrity-Park et al. 2012; Masoodi et al. 2012). MPO is localized within azurophilic granules within neutrophils and is eventually discharged following their activation and degranulation. Furthermore, activation of the inflammatory cells triggers oxidative stress by means of the liberation of reactive oxygen species (ROS) resulting in intestinal tissue injury (Rana et al. 2014; Jena et al. 2012). Contrariwise, oxidative stress and redox signaling up-regulate inflammatory cytokines and attract inflammatory cells via multiple signals including NF-κB activation (Zhang et al. 2019; Öhman and Simrén 2010). Therefore, both oxidative stress and inflammation are linked biological processes contributing to induction of UC.

The present investigation confirmed enhanced oxidative stress through significant elevations of colonic NO content and MPO activity in mice with oxazolone-induced colitis. Virtually, sustained colonic NO generation by iNOS-expressing macrophages plays a central role in the pathogenesis of UC. As NO in combination with superoxide anion yields massive amounts of peroxynitrite radical that inflicts necrosis likely through lipid oxidation and DNA fragmentation (Soufli et al. 2016; Cross and Wilson 2003). The MPO enzyme is a pivotal factor in the induction of oxidative stress in the colon, as it leads to the overproduction of hypochlorous acid, a highly reactive oxidant that exerts cytotoxic effects and contributes to the pathophysiological manifestations of UC (Chami et al. 2021; Yang et al. 2023).

In the present research study, DCA greatly enhanced antioxidant defense in mice with oxazolone colitis, which was reflected by a significant reduction in NO levels along with MPO enzymatic activity. These findings reinforce the premise that the antioxidant properties of DCA are implicated in the alleviation of oxazolone-induced colitis. In accordance, previous reports have described the considerable antioxidant activity DCA in several experimental models (Torres-Cavazos et al. 2020; Zhao et al. 2021; Li et al. 2020). The observed reduction in NO levels after treatment with DCA may be attributed to its suppressive impact on NF-κB, which is responsible for enhancing the transcription of iNOS; the primary contributor to NO production during inflammatory processes (Saed et al. 2011; Mercken et al. 2009).

Interestingly, DCA has undergone extensive research, and multiple clinical studies have suggested that long-term DCA administration is generally safe and well-tolerated for human use, with no hematologic, cardiac, pulmonary, or renal toxicity, when administered under appropriate medical supervision (Abdelmalak et al. 2013; Stacpoole et al. 1998; Khan et al. 2014; Agbenyega et al. 2003). That indicates that DCA is a good candidate for further investigations.

## Conclusion

For the first time, the core targets for management of UC were investigated based on bioinformatics analysis using 3 GEO datasets that revealed the persistence of NFATC1, NLRP3, and IL1B in UC despite treatment with common therapeutic agents. Accordingly, in vivo investigation of the therapeutic efficacy of DCA on oxazolone-induced UC in BALB/c mice revealed its ability to reduce the protein expression level of NLRP3 inflammasome, together with its promotor proteins; NFATC1 and nuclear NF-κB. In addition, the suppressor effect of DCA on NLRP3 was confirmed through its ability to supress linked protein activation, as cleaved caspase-1 expression and the level of IL-1β cytokine, as well as IL-13 level as a predominant inflammatory cytokine in UC. Therefore, DCA could be suggested as a novel and promising candidate for management of UC that merits further safety/toxicological pre-clinical assessment and update of bioavailability/metabolism data prior to clinical investigation.

## Supplementary Information

Below is the link to the electronic supplementary material.

Supplementary file1 (PPTX 2536 KB)

## Data Availability

Enquiries about data availability should be directed to the authors.
