# Correction to: “Management of ulcerative colitis by dichloroacetate: impact on NFATC1/NLRP3/IL1B signaling based on bioinformatics analysis combined with in vivo experimental verification” [Inflammopharmacology (2024) 32:667–682]

**DOI:** 10.1007/s10787-025-02025-0

**Published:** 2025-11-10

**Authors:** Esraa Abdel‑Razek, Heba M. Mahmoud, Amany A. Azouz

**Affiliations:** Pharmacology and Toxicology Department, Facultyof Pharmacy, Beni-Suef University, Beni‑Suef, 62514 Egypt

**Correction to: Inflammopharmacology (2024) 32:667–682**

10.1007/s10787-023-01362-2

The authors regret for the unintended mistake in Fig. 5-panel D, where the images of “DCA” and” Oxzalone + DCA” are identical. The image belongs to Oxazolone + DCA treated group, but it was placed by mistake in DCA control group. This error was due to the large number of colon photos and the non-significant difference of colon length between the two groups. Figure 5 corrigendum presents panel D with the correct image for DCA group.

Some images appear shorter than the data presented in the graph of Fig. 5-panel D due to the folded parts of the colon. The corrigendum of Fig. 5-panel D presents the colon length graph after re-measuring the colons without unfolding. The authors want to emphasize that the corrected results have the same statistical significance and level of significance as those of the 1st measurement of colon length.

**Fig. 5:**
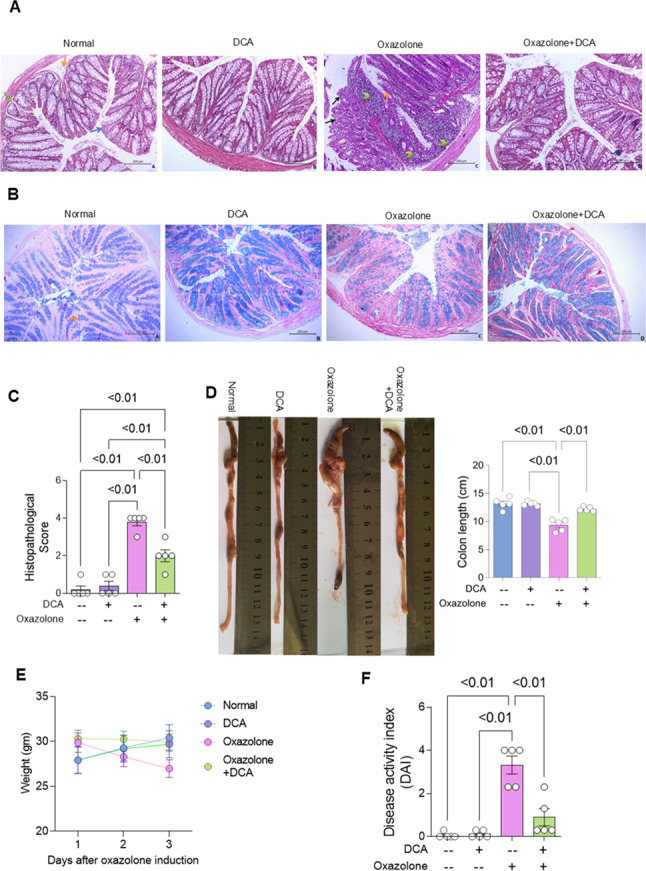
DCA treatment improved colonic morphology and retarded weight loss and disease activity of colitis-induced mouse model. **A** Representative photomicrographs demonstrating colonic sections (× 200) following H&E-staining of colons dissected from normal and UC mice received vehicle or DCA treatment. The normal group showed normal histological structure of the colon and regular arrangement of the lining layers; mucosa (blue arrow), submucosa (orange arrow) and musculosa (green arrow). As well, the normal group received DCA showed normal histopathological features. While colitis group revealed the histopathological features of colitis represented by mucosal hyperplasia (orange arrow), massive focal to diffuse infiltration of mononuclear cells and neutrophils in the lamina propria and submucosa (arrowhead), and mucosal ulcerations (black arrow) in some areas were detected in the colon. Colitis group treated with DCA showed marked improvement of colitis pathology in the form of normal preserved histological colon wall structure, but mild inflammatory cell infiltrations in the lamina propria were still observed. **B** Representative photomicrographs demonstrating colonic sections (× 200) following Alcian blue staining to determine goblet cells of colons dissected from normal and UC mice received vehicle or DCA treatment. Normal group showed intact Alcian blue positive goblet cells in the epithelium and crypts lining (arrow), normal group received DCA revealed abundant goblet cells, while colitis group showed marked reduction in goblet cells, but colitis group treated with DCA showed an almost normal content of goblet cells in colonic section. **C** Histopathological score following H&E staining (*n* = 5 mice for each group). **D–F** Images showing dissected colons from mice receiving vehicle or DCA, mice weights, and disease activity index after oxazolone administration in those mice

The authors confirm that this error does not affect the results or conclusions presented in the study. The authors would like to apologise for any inconvenience caused.

