# Supplementary material for: Management of ulcerative colitis by dichloroacetate: Impact on NFATC1/NLRP3/IL1B signaling based on bioinformatics analysis combined with in vivo experimental verification
Source: Inflammopharmacology. 2023 Oct 30;32(1):667–82. doi: 10.1007/s10787-023-01362-2 (PMC10907436; doi:10.1007/s10787-023-01362-2)
Supplement: Supplementary file 1 — Supplementary file1 (PPTX 2536 KB) [file 10787_2023_1362_MOESM1_ESM.pptx]

## Slide 1
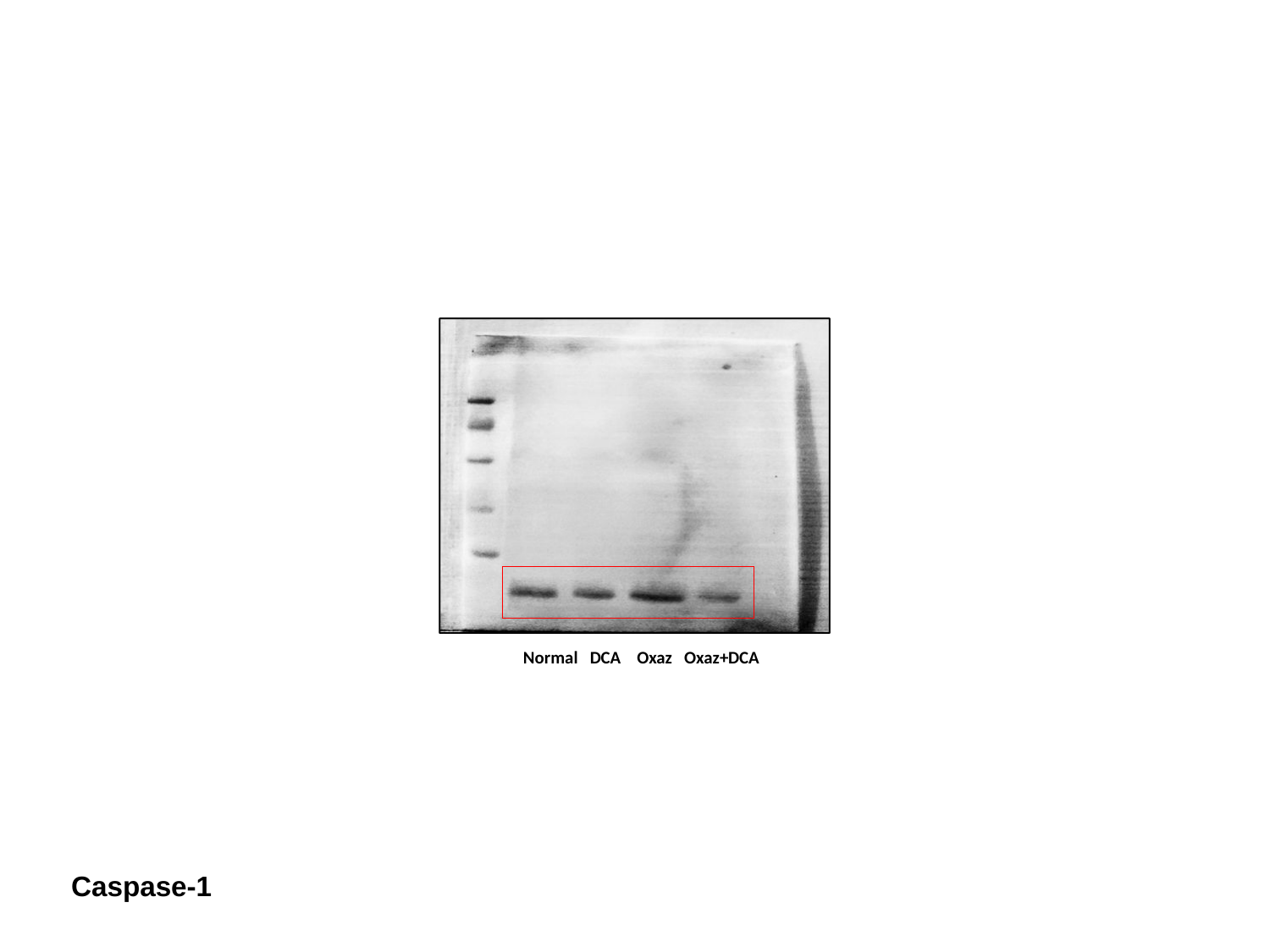

Normal DCA Oxaz Oxaz+DCA
Caspase-1

## Slide 2
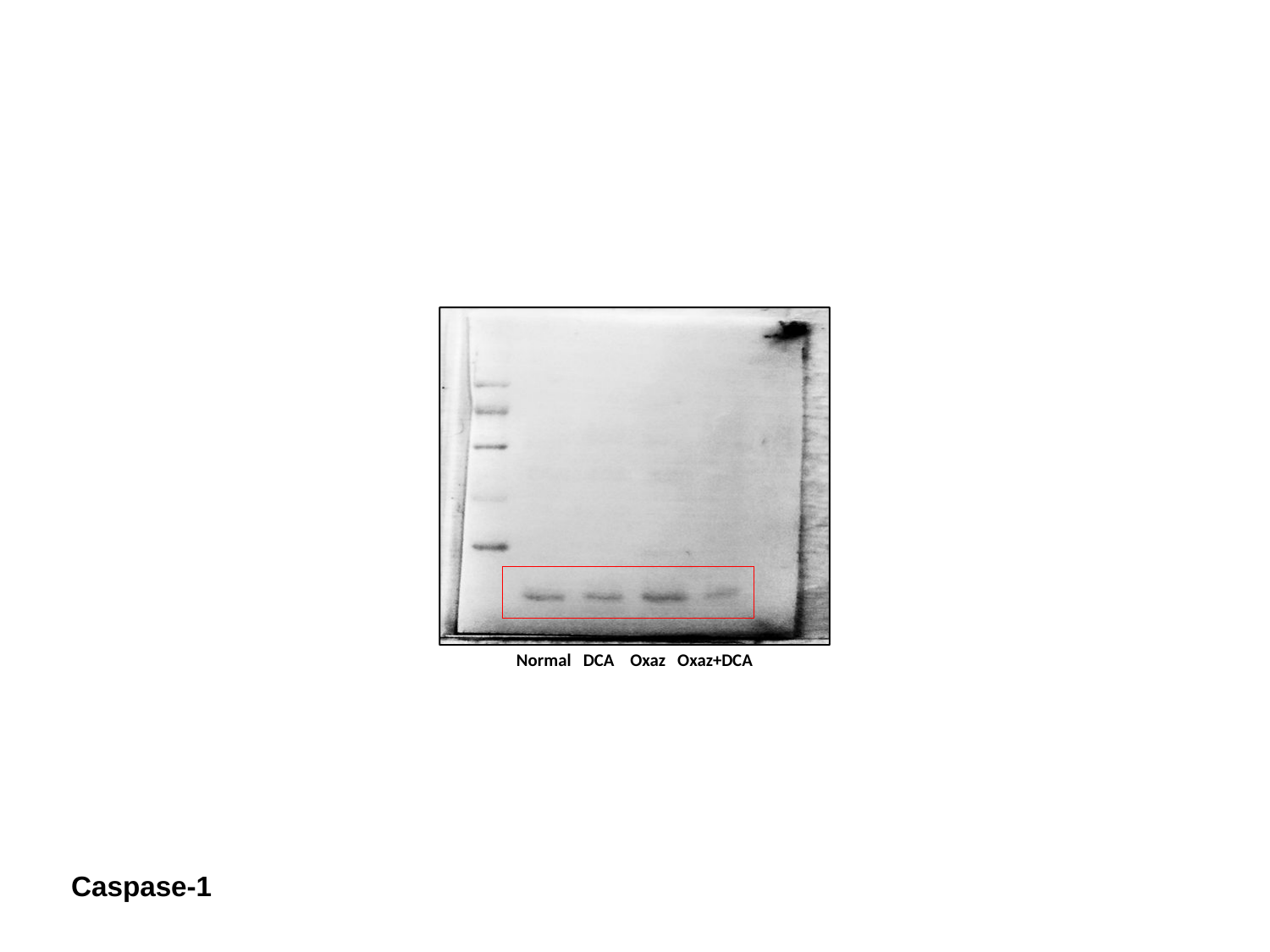

Normal DCA Oxaz Oxaz+DCA
Caspase-1

## Slide 3
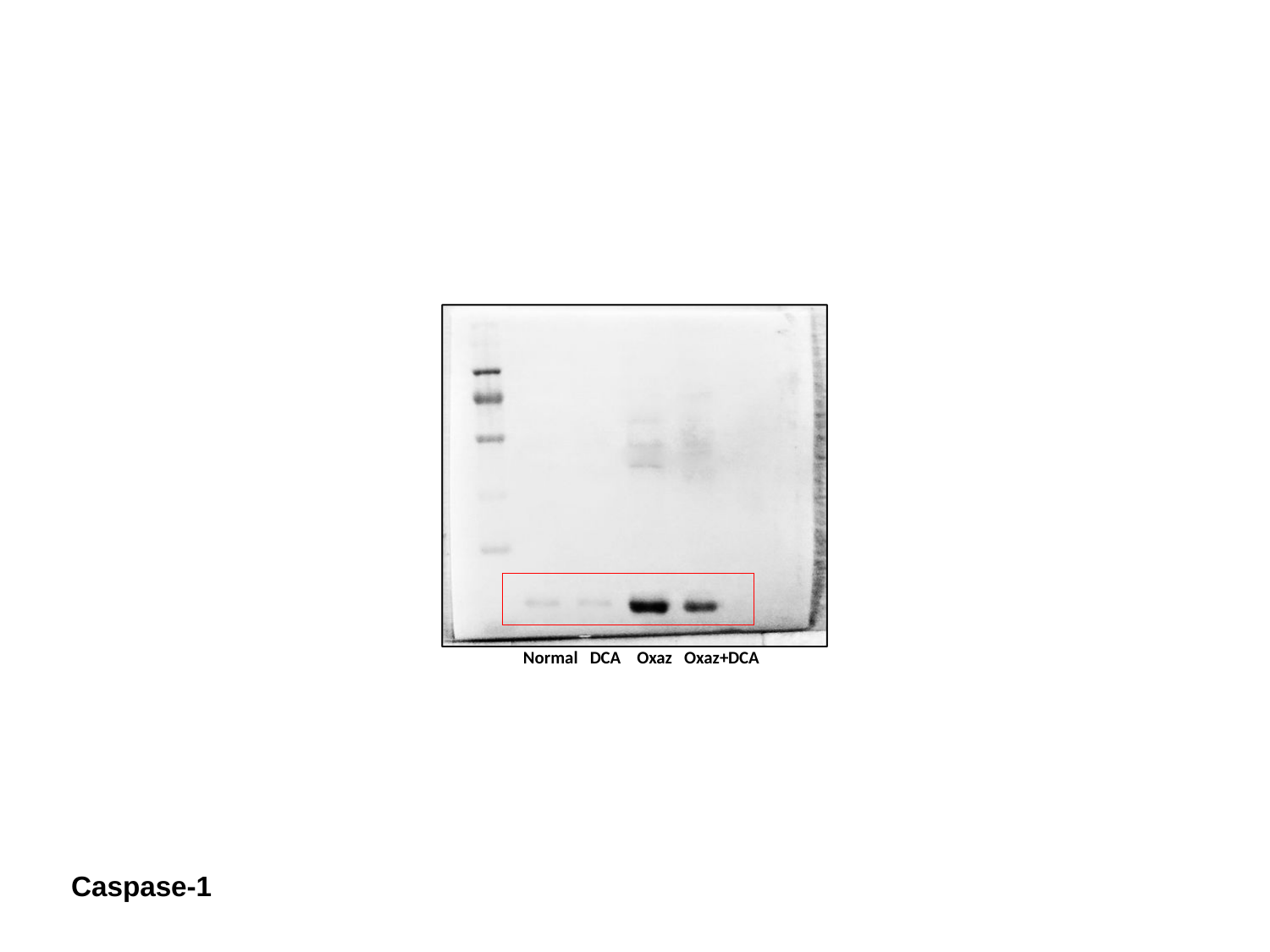

Normal DCA Oxaz Oxaz+DCA
Caspase-1

## Slide 4
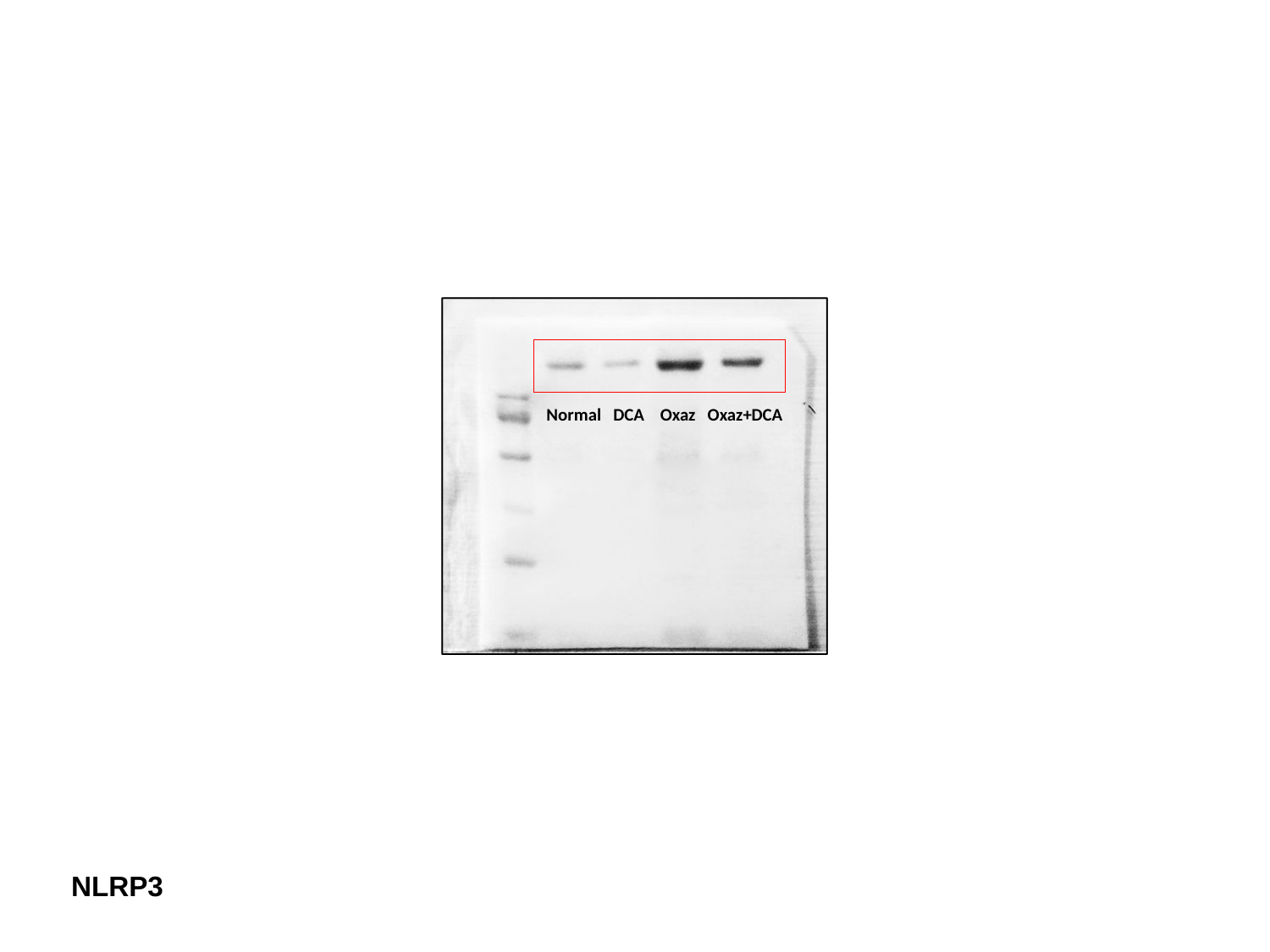

Normal DCA Oxaz Oxaz+DCA
NLRP3

## Slide 5
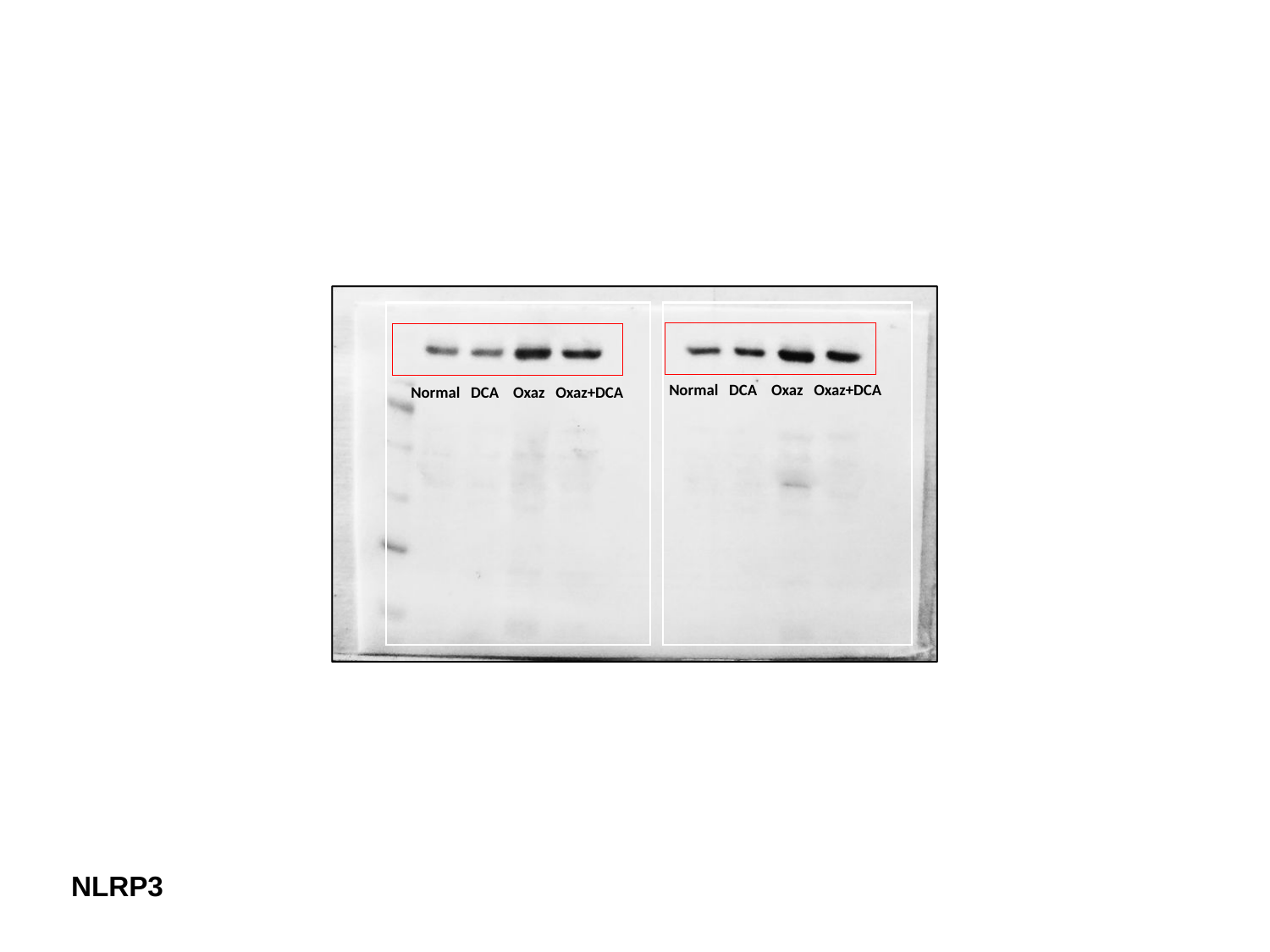

Normal DCA Oxaz Oxaz+DCA
Normal DCA Oxaz Oxaz+DCA
NLRP3

## Slide 6
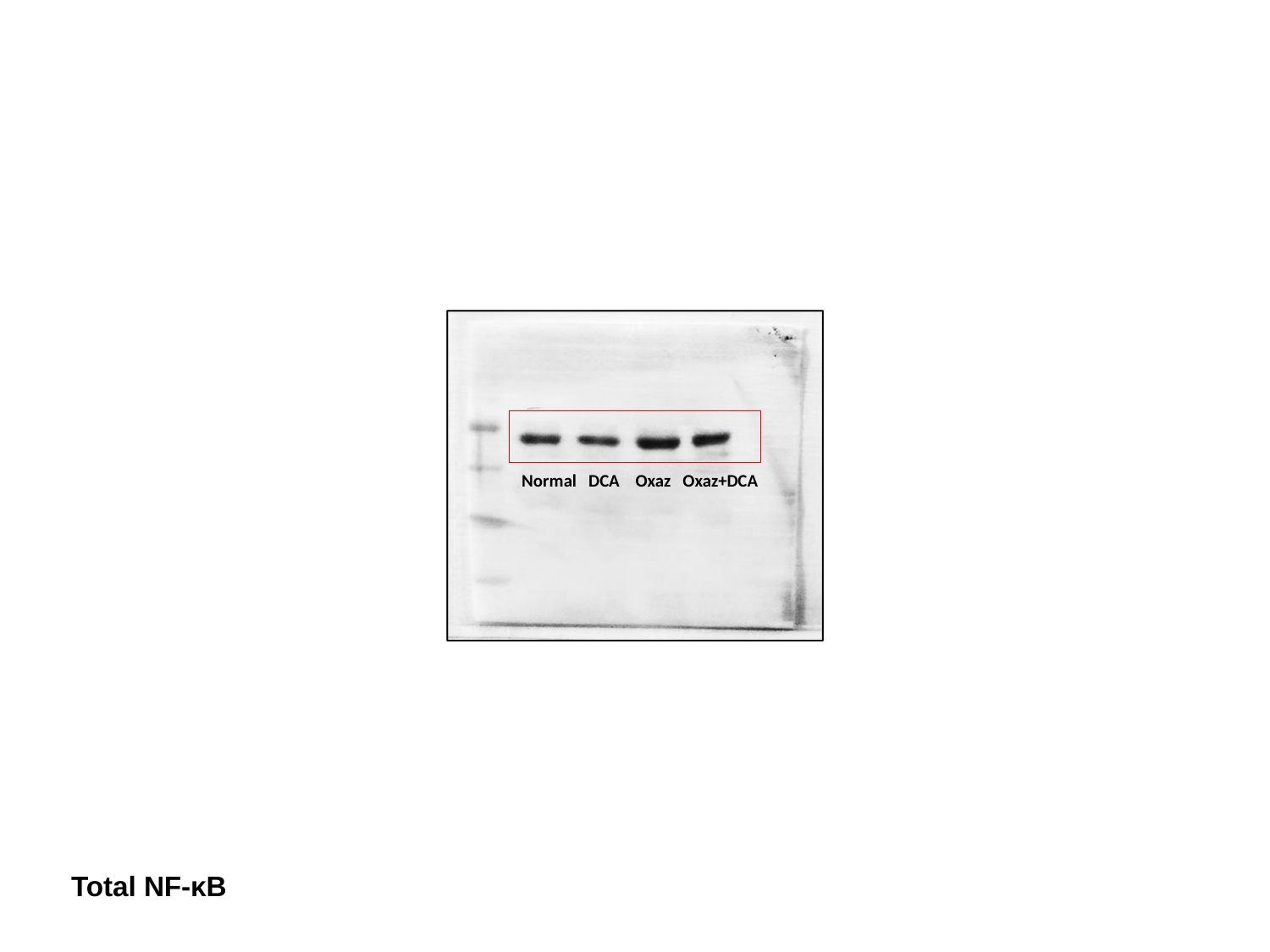

Normal DCA Oxaz Oxaz+DCA
Total NF-κB

## Slide 7
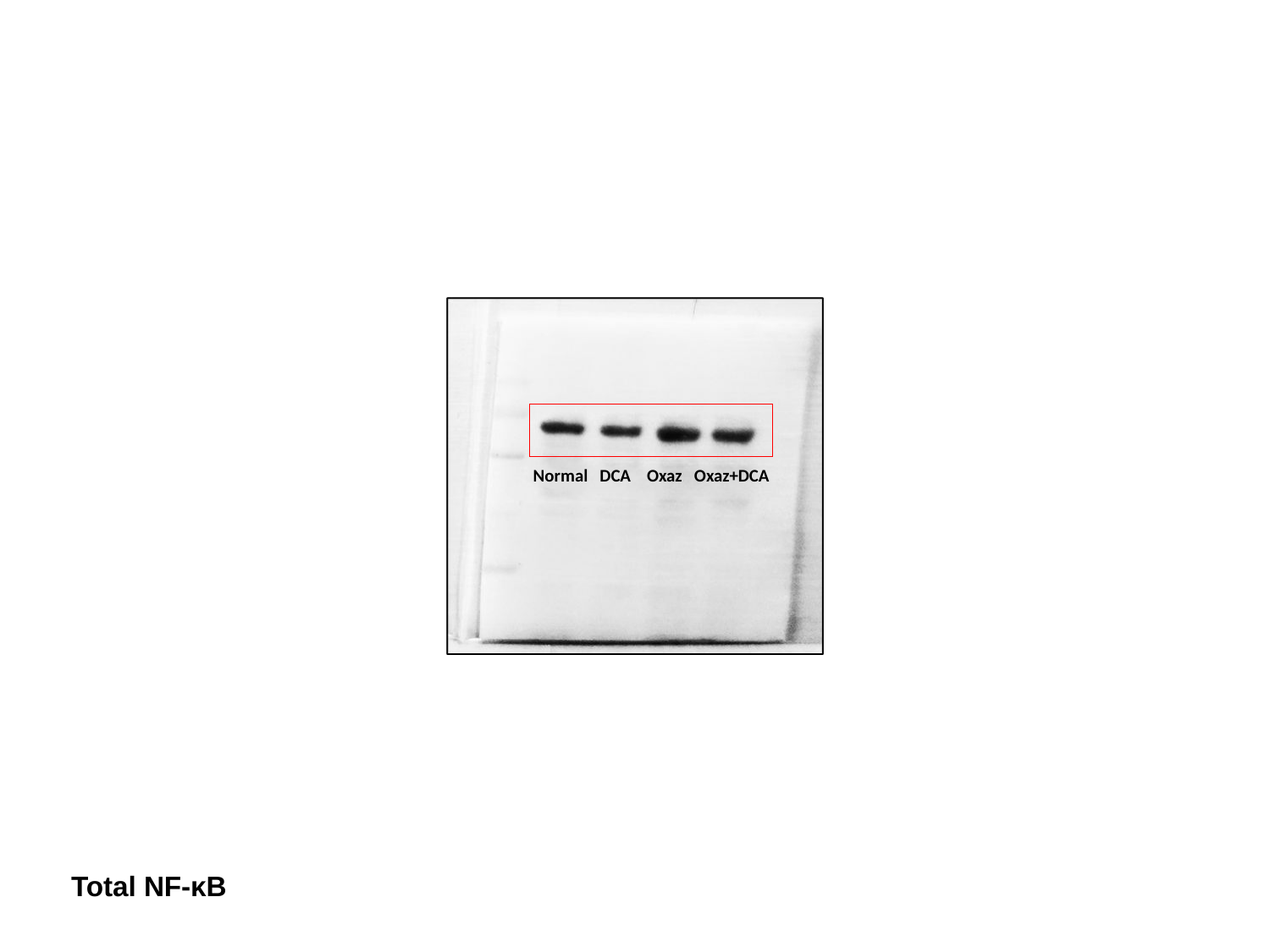

Normal DCA Oxaz Oxaz+DCA
Total NF-κB

## Slide 8
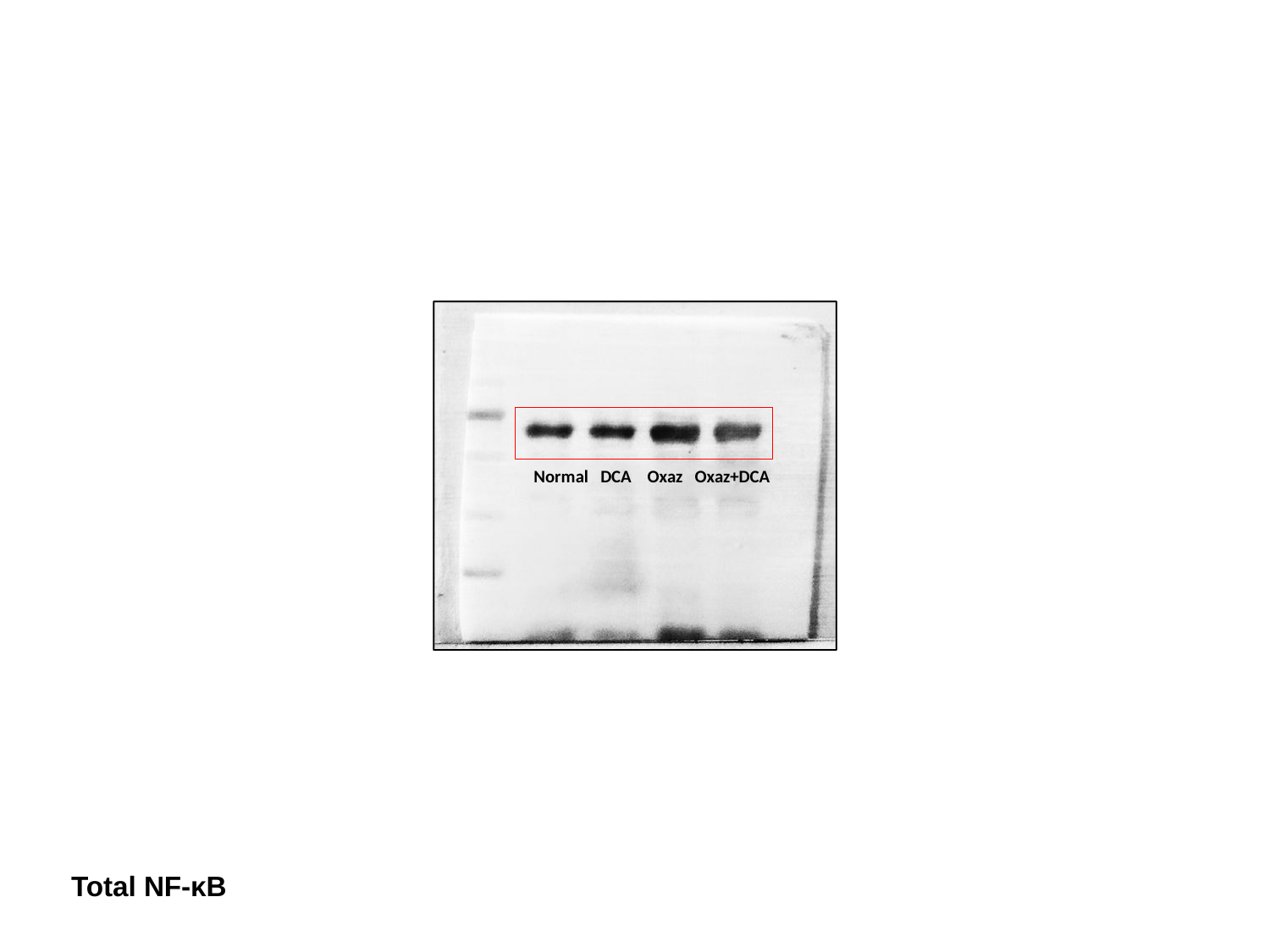

Normal DCA Oxaz Oxaz+DCA
Total NF-κB

## Slide 9
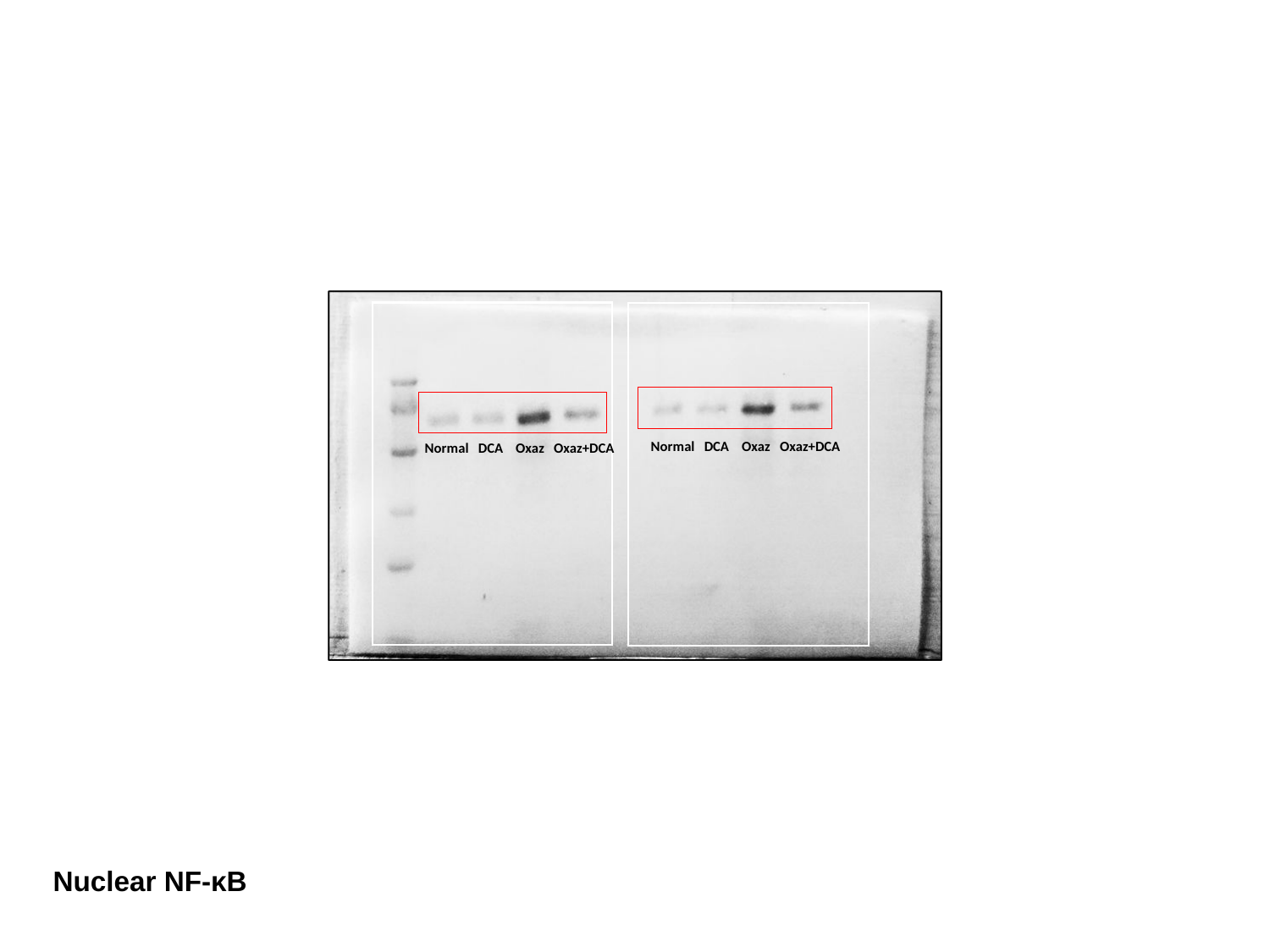

Normal DCA Oxaz Oxaz+DCA
Normal DCA Oxaz Oxaz+DCA
Nuclear NF-κB

## Slide 10
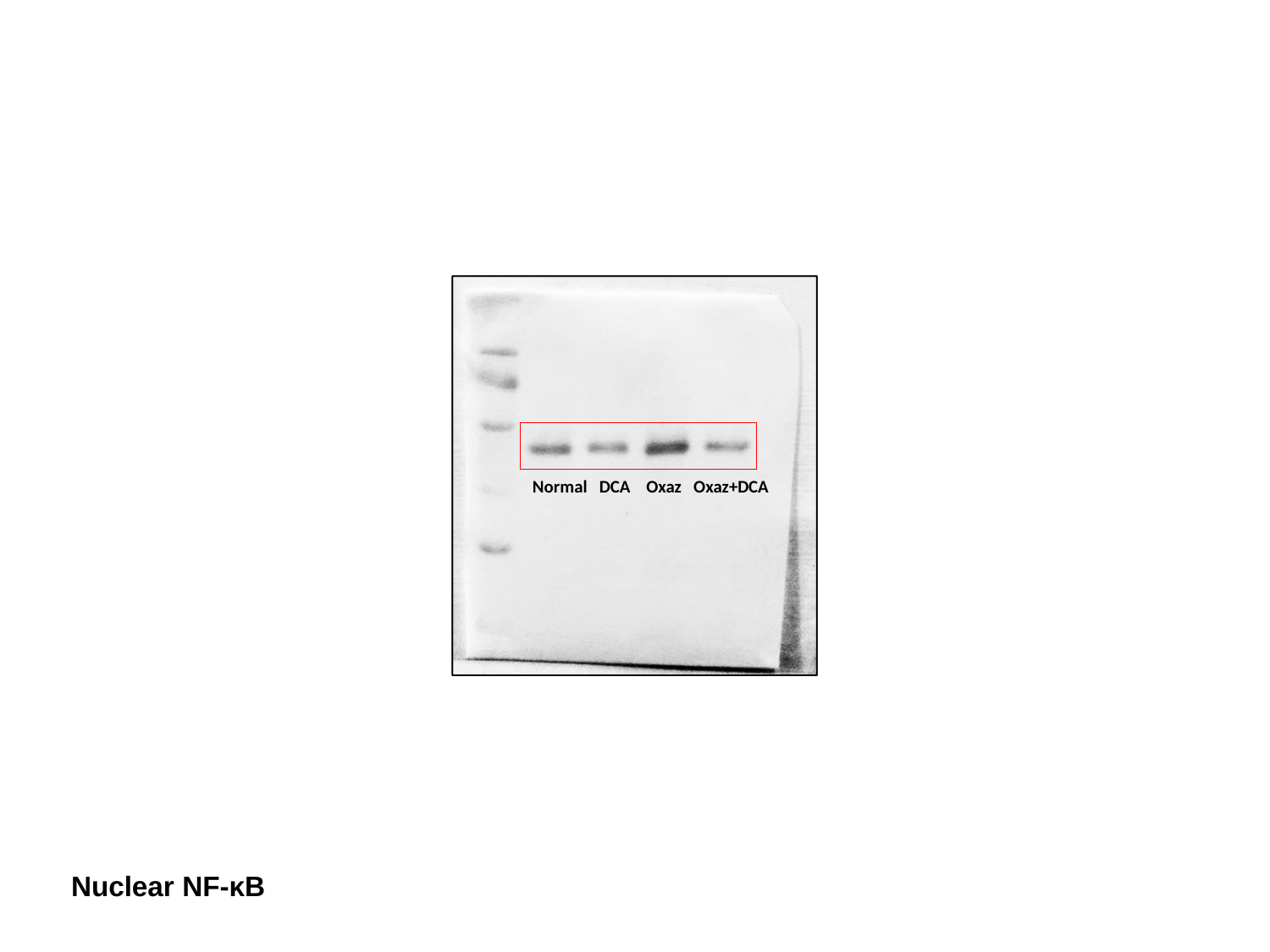

Normal DCA Oxaz Oxaz+DCA
Nuclear NF-κB

## Slide 11
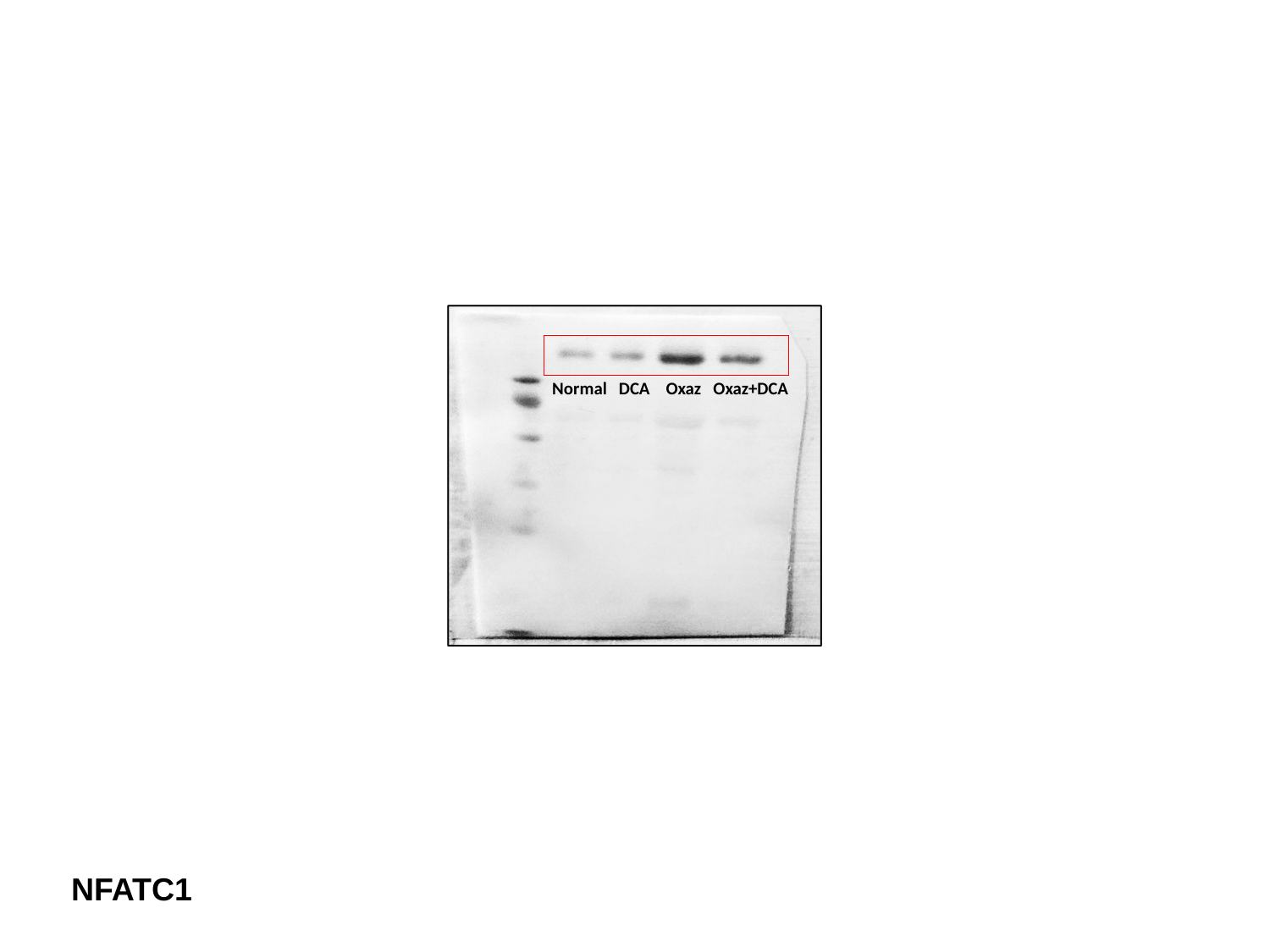

Normal DCA Oxaz Oxaz+DCA
NFATC1

## Slide 12
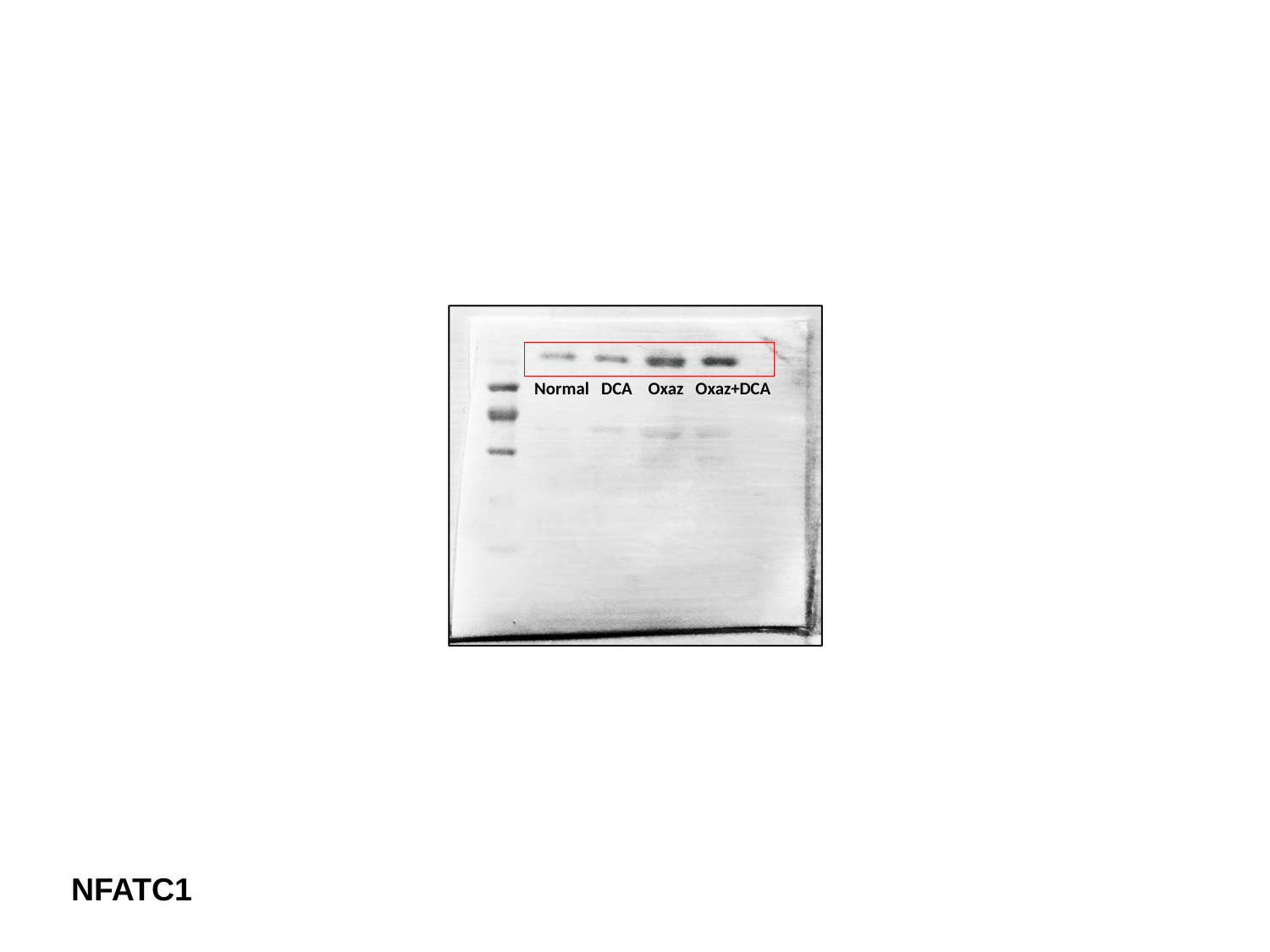

Normal DCA Oxaz Oxaz+DCA
NFATC1

## Slide 13
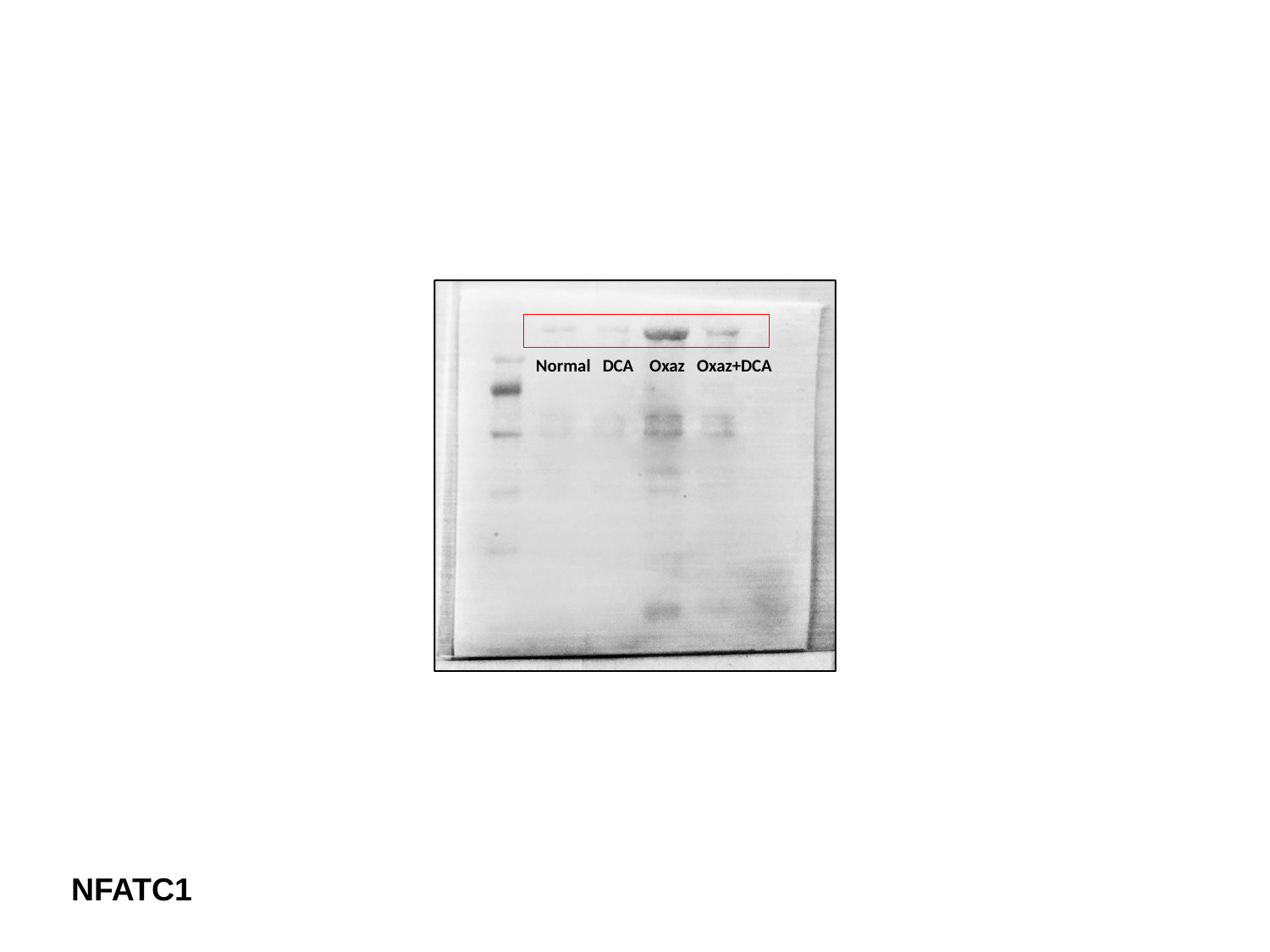

Normal DCA Oxaz Oxaz+DCA
NFATC1

## Slide 14
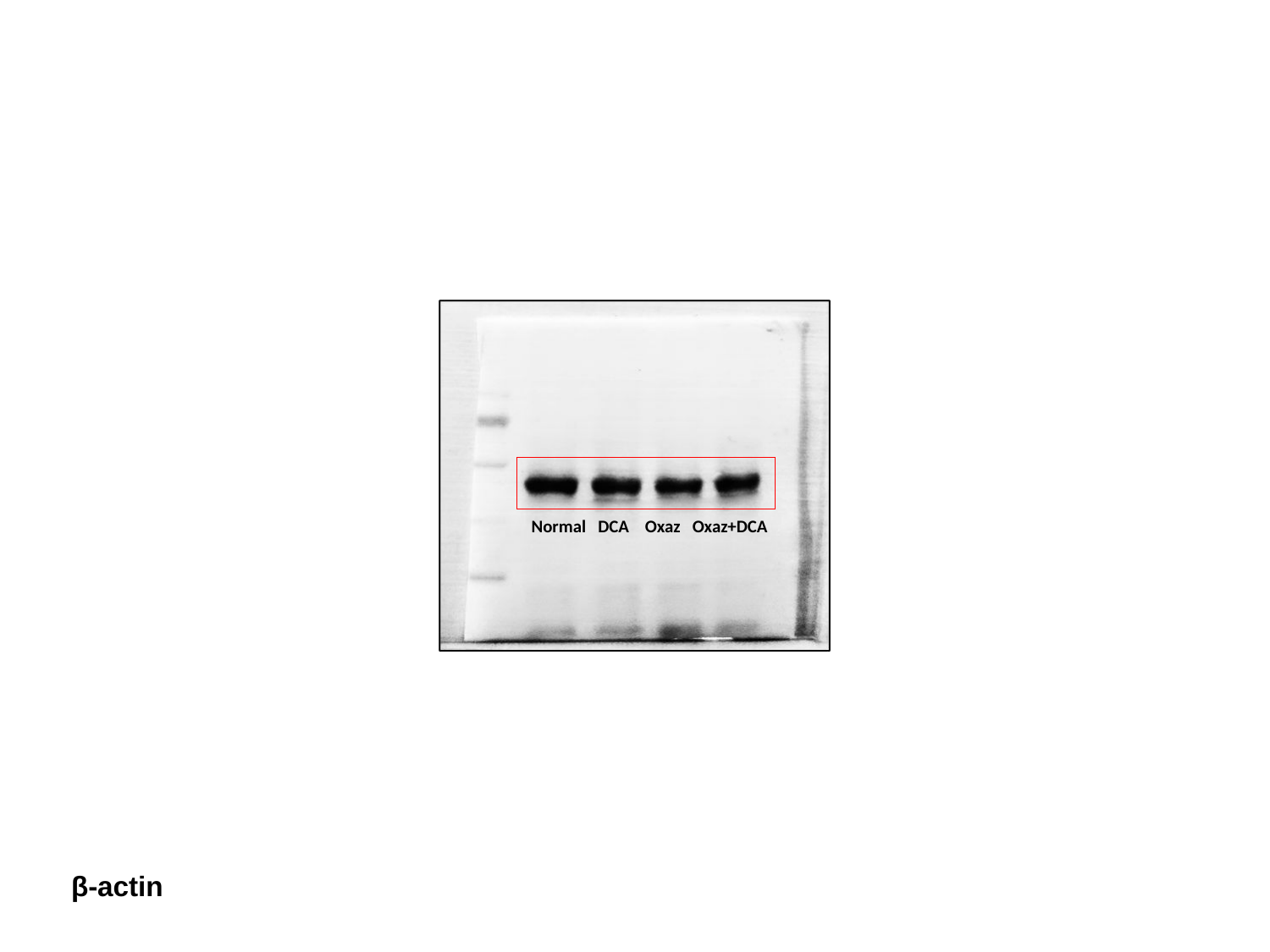

Normal DCA Oxaz Oxaz+DCA
β-actin

## Slide 15
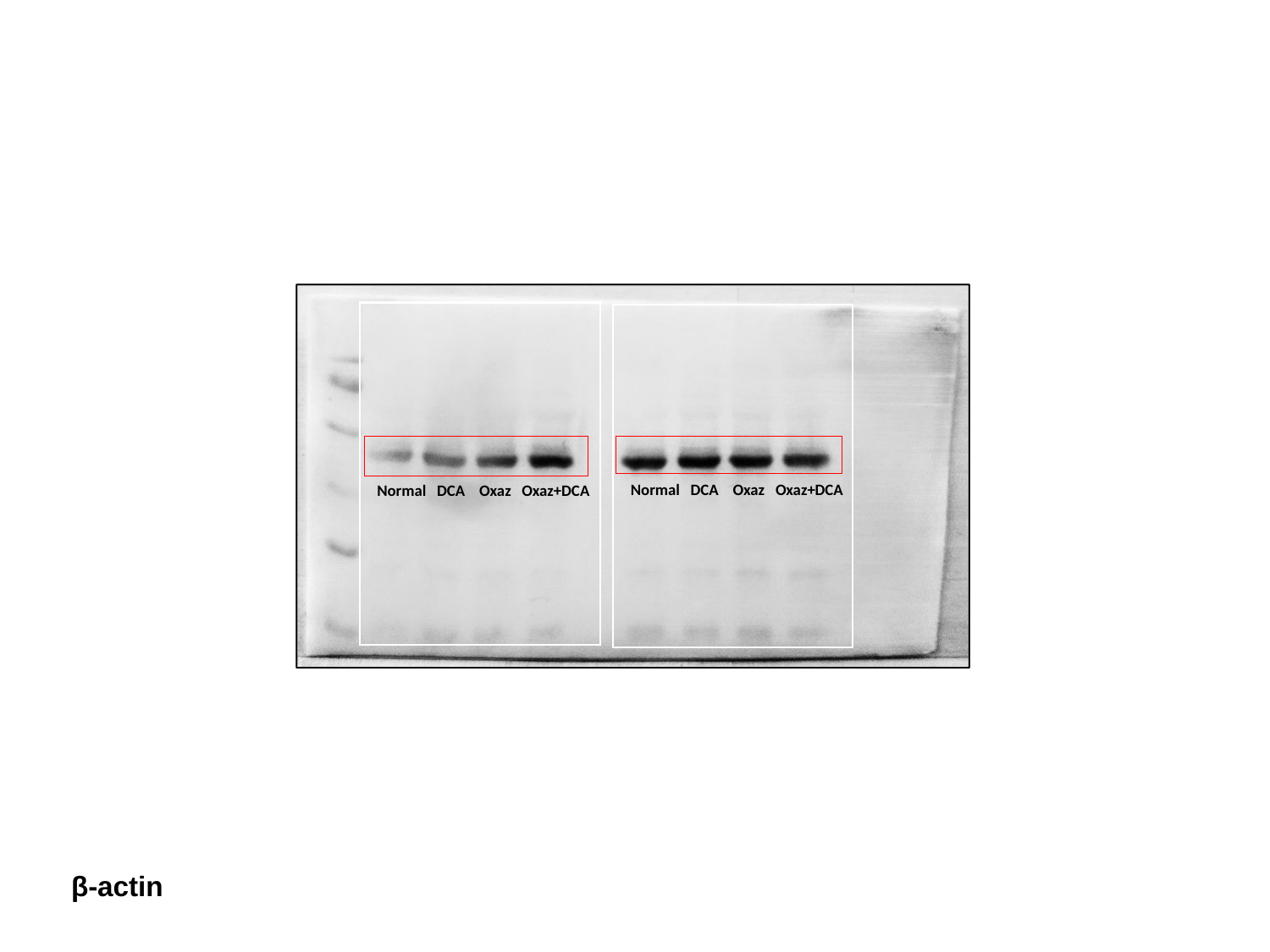

Normal DCA Oxaz Oxaz+DCA
Normal DCA Oxaz Oxaz+DCA
β-actin
